# Supplementary material for: ABO blood group and the risk of aortic disease: a nationwide cohort study
Source: BMJ Open. 2020 Oct 1;10(10):e036040. doi: 10.1136/bmjopen-2019-036040 (PMC7534704; doi:10.1136/bmjopen-2019-036040)
Supplement: Supplementary data [file bmjopen-2019-036040supp001.pdf]

Supplementary table 1. ICD codes used for group classifications

ICD version	Group	Diagnosis	Description
10	1	I710	Aortic dissection
10	1	I711	Thoracic aortic aneurysm, ruptured
10	1	I713	Abdominal aortic aneurysm, ruptured
10	1	I715	Thoracoabdominal aortic aneurysm, ruptured
10	1	I718	Aortic aneurysm with unspecified location, ruptured
10	1	I712	Thoracic aortic aneurysm, non-ruptured
10	1	I714	Abdominal aortic aneurysm, non-ruptured
10	1	I716	Thoracoabdominal aortic aneurysm, non-ruptured
10	1	I719	Aortic aneurysm with unspecified location, non-ruptured
10	2	I710	Aortic dissection
10	3	I711	Thoracic aortic aneurysm, ruptured
10	3	I713	Abdominal aortic aneurysm, ruptured
10	3	I715	Thoracoabdominal aortic aneurysm, ruptured
10	3	I718	Aortic aneurysm with unspecified location, ruptured
10	3	I712	Thoracic aortic aneurysm, non-ruptured
10	3	I714	Abdominal aortic aneurysm, non-ruptured
10	3	I716	Thoracoabdominal aortic aneurysm, non-ruptured
10	3	I719	Aortic aneurysm with unspecified location, non-ruptured
9	1	4410	Aortic dissection
9	1	4411	Thoracic aortic aneurysm, ruptured
9	1	4413	Abdominal aortic aneurysm, ruptured
9	1	4416	Thoracoabdominal aortic aneurysm, ruptured
9	1	4415	Aortic aneurysm with unspecified location, ruptured
9	1	4412	Thoracic aortic aneurysm, non-ruptured
9	1	4414	Abdominal aortic aneurysm, non-ruptured
9	1	4417	Thoracoabdominal aortic aneurysm, non-ruptured
9	1	4419	Aortic aneurysm with unspecified location, non-ruptured
9	2	4410	Aortic dissection
9	3	4411	Thoracic aortic aneurysm, ruptured
9	3	4413	Abdominal aortic aneurysm, ruptured
9	3	4416	Thoracoabdominal aortic aneurysm, ruptured
9	3	4415	Aortic aneurysm with unspecified location, ruptured
9	3	4412	Thoracic aortic aneurysm, non-ruptured
9	3	4414	Abdominal aortic aneurysm, non-ruptured

9	3	4417	Thoracoabdominal aortic aneurysm, non-ruptured
9	3	4419	Aortic aneurysm with unspecified location, non-ruptured
8	1	4410	Aortic dissection
8	1	4411	Thoracic aortic aneurysm
8	1	4412	Abdominal aortic aneurysm
8	1	4419	Aortic aneurysm with unspecified location, non-ruptured
8	2	4410	Aortic dissection
8	3	4411	Thoracic aortic aneurysm
8	3	4412	Abdominal aortic aneurysm
8	3	4419	Aortic aneurysm with unspecified location, non-ruptured
7	1	45100	Aortic dissection
7	1	45110	Aortic dissection

Supplementary table 2. Group description

Group	Group name	Description
1	Aortic disease	Aortic aneurysm and aortic dissection
2	Aortic dissection	Aortic dissection
3	Aortic aneurysm	Non-ruptured or ruptured aortic aneurysm

Supplementary table 3. Regression estimates and corresponding Incidence Rate Ratios, presented separately for the donor and recipient cohorts.

Group	Variable	Donors' Estimates Regression Estimate	Donors' Standard Error	Donors' IRR (95%CI)	Patients' Estimates Regression Estimate	Patients' Standard Error	Patients' IRR (95%CI)
Aneurysm and Dissection							
	Intercept	-88.131	70.894		-890.153	167.331	
	Blood Group						
	A	0.003	0.031	1.00 (0.94-1.06)	0.018	0.017	1.02 (0.98-1.05)
	AB	-0.058	0.065	0.94 (0.83-1.07)	0.031	0.038	1.04 (0.96-1.12)
	B	-0.072	0.049	0.93 (0.85-1.03)	-0.095	0.029	0.90 (0.85-0.96)
	O	0.000	.	1.00 (.-.)	0.000	.	1.00 (.-.)
	Non-o	-0.016	0.029	0.98 (0.93-1.04)	0.001	0.016	1.00(0.97-1.03)
	Sex						
	Female	1.250	0.040	3.49 (3.23-3.78)	1.115	0.017	3.05(2.95-3.15)
	Male	0.000	.	1.00 (.-.)	0.000	.	1.00 (.-.)
	Age (spline)						
	Level 1	0.000	.	1.00 (.-.)	0.000	.	1.00 (.-.)
	Level 2	0.088	0.010	1.09 (1.07-1.11)	0.033	0.011	1.03(1.01-1.06)
	Level 3	0.003	0.001	1.00 (1.00-1.00)	0.004	0.001	1.00(1.00-1.01)
	Level 4	-0.004	0.002	1.00 (0.99-1.00)	-0.006	0.002	0.99(0.99-1.00)
	Level 5	-0.003	0.001	1.00 (0.99-1.00)	-0.001	0.001	1.00(1.00-1.00)

	Year (spline)						
	Level 1	0.000	.	1.00 (.-.)	0.000	.	1.00 (.-.)
	Level 2	0.037	0.036	1.04 (0.97-1.11)	0.443	0.084	1.56(1.32-1.84)
	Level 3	-0.016	0.007	0.98 (0.97-1.00)	-0.041	0.010	0.96(0.94-0.98)
	Level 4	0.053	0.014	1.05 (1.03-1.08)	0.075	0.017	1.08(1.04-1.11)
	Level 5	-0.057	0.010	0.94 (0.93-0.96)	-0.047	0.009	0.95(0.94-0.97)
Aortic Dissection							
	Intercept	-59.428	114.105		-1112.308	397.726	
	Blood Group						
	A	-0.131	0.071	0.89 (0.77-1.01)	-0.052	0.047	0.98 (0.89-1.08)
	AB	-0.128	0.150	0.93 (0.70-1.23)	0.146	0.097	1.16 (0.95-1.41)
	B	-0.123	0.111	0.88 (0.71-1.09)	0.059	0.074	1.05 (0.91-1.22)
	O	0.000	.	1.00 (.-.)	0.000	.	1.00 (.-.)
	Non-o	-0.129	0.066	0.89 (0.78-1.01)	-0.016	0.044	1.01 (0.92-1.10)
	Sex						
	Female	0.000	.	1.00 (.-.)	0.000	.	1.00 (.-.)
	Male	0.956	0.083	2.60 (2.21-3.06)	0.745	0.045	2.11(1.93-2.30)
	Age (spline)						
	Level 1	0.000	.	1.00 (.-.)	0.000	.	1.00 (.-.)
	Level 2	0.135	0.019	1.14 (1.10-1.19)	0.041	0.019	1.04(1.00-1.08)
	Level 3	-0.002	0.002	1.00 (0.99-1.00)	0.001	0.002	1.00(1.00-1.00)

	Level 4	0.004	0.004	1.00 (1.00-1.01)	-0.002	0.003	1.00(0.99-1.00)
	Level 5	-0.004	0.003	1.00 (0.99-1.00)	-0.001	0.002	1.00(0.99-1.00)
	Year (spline)						
	Level 1	0.000	.	1.00 (.-.)	0.000	.	1.00 (.-.)
	Level 2	0.021	0.058	1.02 (0.91-1.14)	0.554	0.201	1.74(1.18-2.58)
	Level 3	-0.017	0.011	0.98 (0.96-1.01)	-0.056	0.024	0.95(0.90-0.99)
	Level 4	0.058	0.024	1.06 (1.01-1.11)	0.098	0.041	1.10(1.02-1.20)
	Level 5	-0.067	0.018	0.94 (0.90-0.97)	-0.058	0.020	0.94(0.91-0.98)
Aortic Aneurysm							
	Intercept	-139.788	91.425		-861.716	185.020	
	Blood Group						
	A	0.029	0.033	1.02 (0.96-1.09)	0.020	0.018	1.02 (0.98-1.05)
	AB	-0.033	0.069	0.97 (0.85-1.11)	0.030	0.039	1.04 (0.97-1.13)
	B	-0.048	0.051	0.95 (0.86-1.05)	-0.114	0.030	0.89 (0.84-0.94)
	O	0.000	.	1.00 (.-.)	0.000	.	1.00 (.-.)
	Non-o	0.010	0.031	1.00 (0.95-1.07)	0.000	0.017	1.00(0.96-1.03)
	Sex						
	Female	1.299	0.043	3.67 (3.37-3.99)	1.158	0.018	3.18(3.07-3.30)
	Male	0.000	.	1.00 (.-.)	0.000	.	1.00 (.-.)
	Age (spline)						
	Level 1	0.000	.	1.00 (.-.)	0.000	.	1.00 (.-.)
	Level 2	0.074	0.011	1.08 (1.05-1.10)	0.035	0.013	1.04(1.01-1.06)

Level 3	0.004	0.001	1.00 (1.00-1.01)	0.004	0.001	1.00(1.00-1.01)
Level 4	-0.007	0.002	0.99 (0.99-1.00)	-0.007	0.002	0.99(0.99-1.00)
Level 5	-0.001	0.001	1.00 (1.00-1.00)	0.000	0.001	1.00(1.00-1.00)
Year (spline)						
Level 1	0.000	.	1.00 (.-.)	0.000	.	1.00 (.-.)
Level 2	0.063	0.046	1.06 (0.97-1.17)	0.428	0.093	1.53(1.28-1.84)
Level 3	-0.019	0.008	0.98 (0.97-1.00)	-0.038	0.011	0.96(0.94-0.98)
Level 4	0.058	0.017	1.06 (1.03-1.09)	0.071	0.019	1.07(1.03-1.11)
Level 5	-0.062	0.011	0.94 (0.92-0.96)	-0.047	0.009	0.95(0.94-0.97)

5
