# Supplementary material for: ABO blood group and the risk of aortic disease: a nationwide cohort study
Source: BMJ Open. 2020 Oct 1;10(10):e036040. doi: 10.1136/bmjopen-2019-036040 (PMC7534704; doi:10.1136/bmjopen-2019-036040)
Supplement: Author's manuscript [file bmjopen-2019-036040.draft_revisions.pdf]

BMJ Open

BMJ Open is committed to open peer review. As part of this commitment we make the peer review history of every article we publish publicly available.

When an article is published we post the peer reviewers' comments and the authors' responses online. We also post the versions of the paper that were used during peer review. These are the versions that the peer review comments apply to.

The versions of the paper that follow are the versions that were submitted during the peer review process. They are not the versions of record or the final published versions. They should not be cited or distributed as the published version of this manuscript.

BMJ Open is an open access journal and the full, final, typeset and author-corrected version of record of the manuscript is available on our site with no access controls, subscription charges or pay-per-view fees (http://bmjopen.bmj.com).

If you have any questions on BMJ Open's open peer review process please email info.bmjopen@bmj.com

BMJ Open

ABO blood group and the risk of aortic disease - A nationwide cohort study

Journal:	*BMJ Open*
Manuscript ID	bmjopen-2019-036040
Article Type:	Original research
Date Submitted by the Author:	01-Dec-2019

Complete List of Authors:	Zindovic, Igor; Department of Cardiothoracic Surgery, Clinical Sciences, Lund University, Skane University Hospital<br>Edgren, Gustaf; Karolinska Universitetssjukhuset, Department of Medicine, Solna, Clinical Epidemiology Division, Karolinska Institutet, Stockholm, Sweden; Department of Cardiology, Södersjukhuset, Stockholm, Sweden<br>Nozohoor, Shahab; Department of Cardiothoracic Surgery, Clinical Sciences, Lund University, Skane University Hospital<br>Majeed, Ammar; Department of Medicine, Solna, Clinical Epidemiology Division, Karolinska Institutet, Stockholm, Sweden; Department of Gastroenterology, The Alfred Health, Melbourne, Victoria, Australia

Keywords:	EPIDEMIOLOGY, Thoracic surgery < SURGERY, Bleeding disorders & coagulopathies < HAEMATOLOGY

SCHOLARONE™
Manuscripts

BMJ

I, the Submitting Author has the right to grant and does grant on behalf of all authors of the Work (as defined in the below author licence), an exclusive licence and/or a non-exclusive licence for contributions from authors who are: i) UK Crown employees; ii) where BMJ has agreed a CC-BY licence shall apply, and/or iii) in accordance with the terms applicable for US Federal Government officers or employees acting as part of their official duties; on a worldwide, perpetual, irrevocable, royalty-free basis to BMJ Publishing Group Ltd ("BMJ") its licensees and where the relevant Journal is co-owned by BMJ to the co-owners of the Journal, to publish the Work in this journal and any other BMJ products and to exploit all rights, as set out in our licence.

The Submitting Author accepts and understands that any supply made under these terms is made by BMJ to the Submitting Author unless you are acting as an employee on behalf of your employer or a postgraduate student of an affiliated institution which is paying any applicable article publishing charge ("APC") for Open Access articles. Where the Submitting Author wishes to make the Work available on an Open Access basis (and intends to pay the relevant APC), the terms of reuse of such Open Access shall be governed by a Creative Commons licence – details of these licences and which Creative Commons licence will apply to this Work are set out in our licence referred to above.

Other than as permitted in any relevant BMJ Author's Self Archiving Policies, I confirm this Work has not been accepted for publication elsewhere, is not being considered for publication elsewhere and does not duplicate material already published. I confirm all authors consent to publication of this Work and authorise the granting of this licence.

For peer review only

ABO blood group and the risk of aortic disease - A nationwide cohort study

Igor Zindovic MD PhD[1], Gustaf Edgren MD PhD[2,3], Shahab Nozohoor MD PhD[1], Ammar Majeed MD PhD[2,4,5]

[1]Lund University, Skåne University Hospital, Department of Clinical Sciences, Department of Cardiothoracic Surgery, Lund, Sweden; [2]Department of Medicine, Solna, Clinical Epidemiology Division, Karolinska Institutet, Stockholm, Sweden; [3]Department of Cardiology, Södersjukhuset, Stockholm, Sweden; [4]Department of Gastroenterology, The Alfred Health, Melbourne, Victoria, Australia; [5]Central Clinical School, Monash University, Melbourne, Victoria, Australia.

Corresponding author: Igor Zindovic, MD

Department of Cardiothoracic Surgery

Skåne University Hospital, SE-221 85 Lund, Sweden

Phone: +46 46-171000; E-mail: igor.zindovic@med.lu.se

Co-author contact information:

Gustaf.Edgren@ki.se; Shahab.Nozohoor@med.lu.se; Ammar.Majeed@ki.se

For peer review only

ABSTRACT

Objectives: To analyze the association between ABO blood group and aortic disease using data on blood donors and transfused patients from Sweden.

Design: This was a retrospective study using data from the Swedish portion of the SCANDAT2 registry. The association between ABO blood group and aortic disease was analyzed using log-linear Poisson regression models and presented as incidence rate ratios (IRR:s).

Setting: Swedish population-based study.

Participants: The study cohort consisted of 1 164 561 Swedish blood donors and 961 637 transfused patients with a combined follow-up time of 29 390 649 person-years.

Primary and secondary outcome measures: Incidence rate ratios of aortic events (*i.e.* aortic aneurysms and/or aortic dissections) in relation to patient blood group.

Results: A total of 20 684 aortic events occurred during the study period. Non-O donors and patients had similar incidence of aortic events when compared to blood group O donors and patients with an IRR of 0.98 (95% confidence interval [CI], 0.93-1.04) and 1.00 (95% CI, 0.97-1.03), respectively. There were no differences between non-O and blood group O individuals when aortic dissections and aortic aneurysms were analyzed separately. Blood group B conferred a lower risk of aortic aneurysms in the patient cohort when compared to blood group O (IRR, 0.90; 95% CI, 0.85-0.96).

Conclusions: In the present study, there were no statistically significant associations between ABO blood group and the risk of aortic disease. A possible protective effect of blood group B was observed in the patient cohort but this finding requires further investigation.

For peer review only

For peer review only - http://bmjopen.bmj.com/site/about/guidelines.xhtml

STRENGTHS AND LIMITATIONS OF THIS STUDY

- Data were collected from a quality registry covering all blood donations and transfusions 1968 to 2012.
- This study is strengthened by complete follow up of the study cohort.
- This is by far the largest study assessing the association between blood groups and aortic disease.
- The study is limited by donors representing a selected cohort of healthy individuals, whereas the patient cohort has a higher prevalence of co-morbidities compared to the general population.
- Another limitation is that detailed information on co-morbidities of individuals in the study population was not known.

For peer review only

For peer review only - http://bmjopen.bmj.com/site/about/guidelines.xhtml

INTRODUCTION

Aortic aneurysm and dissection are potentially life-threatening conditions that share common risk factors—age, hypertension, atherosclerosis, and smoking—with other types of cardiovascular disease (1-3). The association between ABO blood groups and cardiovascular disease and venous thromboembolism (VTE) has been widely reported, but the pathophysiological mechanisms are not fully understood (4-8).

Few studies have examined the association between ABO blood group and aortic disease (9-11). In an analysis of 504 patients with abdominal aortic aneurysm (AAA), Viklander *et. al.* did not find a significant difference in distribution of blood groups between patients with AAA or ruptured AAA compared to a population-based control group (9). Fatic *et. al.* on the other hand, demonstrated that a significantly higher proportion of AAA patients had blood group O compared to a control group consisting of blood donors from the general population of Montenegro (10). Available research is limited by the small size of the studies, conflicting results and the omission of patients with aortic dissection, warranting further examination of the relationship between blood groups and aortic disease.

The aim of the present study was to evaluate the association between ABO blood group and aortic disease using data from a large cohort of Swedish individuals derived from the Scandinavian Donations and Transfusions (SCANDAT2) database (13).

For peer review only

MATERIAL AND METHODS

Data source

The study was based on the Scandinavian Donations and Transfusions database (SCANDAT2). This is a computerized, combined donation and transfusion register using data on more than 1.6 million blood donors and 2 million recipients of blood products in Sweden from 1968 to 2012 and in Denmark from 1981 to 2012 (12, 13). Using the unique personal identity numbers assigned to all residents of the two countries, SCANDAT2 is linked to national population, death, and migration registers, allowing for the complete follow-up of all individuals in the register up to and including December 31, 2012 (14). The database is also linked to the national inpatient, outpatient, cause of death and cancer registries with information on registered diagnoses coded as the International Classification of Diseases (ICD) codes, versions 6-10.

Study Design

This was a retrospective data analysis based on individuals included in the SCANDAT2 register. For the purpose of these analyses, we only used data from the Swedish portion of the SCANDAT2 database and restricted the analysis to donors and patients who donated or received their first blood product after January 1, 1970, when the patient register in Sweden had good national coverage. Donors and recipients with a documented blood group in SCANDAT2, who had given or received at least one blood product between January 1, 1970 and December 31, 2012, were included in the study. We used a set of ICD codes to identify donors and recipients who were diagnosed with either aortic aneurysm or aortic dissection (Supplementary Table 1) based on information from the patient register. The different ICD codes were grouped into 2 different disease groups based on etiological factors or disease characteristics (Supplementary Table 2). Recognizing that aortic aneurysm and dissection share common risk factors and disease characteristics, we created a third group that encompassed all ICD codes for aortic aneurysm and dissection. Donors and recipients who had a diagnosis of aortic aneurysm and/or dissection prior to their first donation or transfusion were excluded from the study. In the present study, we used the term “aortic disease” to reflect either aortic aneurysm or aortic dissection and the diagnosis of an aortic aneurysm or aortic dissection as an “aortic event”.

For peer review only - http://bmjopen.bmj.com/site/about/guidelines.xhtml

Patient and public involvement

Patients or the public were not involved in the design, or conduct, or reporting, or dissemination plans of the research presented in this work.

Statistical analysis

Recognizing differences in demographics and comorbidities, we created separate cohorts for the donors and the recipients. In the analysis for the donor cohort, we followed the donors from the date of first electronically recorded blood donation in SCANDAT2 or the study start date (January 1, 1970), whichever came last. For the transfusion recipient cohort, individuals were followed from the start of the study (January 1, 1970) or 365 days after their first electronically recorded blood transfusion in SCANDAT2, whichever came last. We delayed the patients' entry into the study by one year after their first transfusion to reduce the risk of reverse causation bias (*i.e.* when the indication for their first transfusion would somehow be related to the subsequent occurrence of an aortic event). All study participants were followed up until either the date of diagnosis of aortic aneurysm or dissection, emigration, death or end of follow-up (December 31, 2012), whichever came first. As an individual could be diagnosed with more than one type of aortic aneurysm or have an aneurysm and dissection diagnosed at different time points during their lifetime, we allowed for the same individual to contribute time at risk and events into more than one disease group based on their diagnoses.

Baseline characteristics are presented as means and standard deviations for continuous variables, and frequencies and percentages for categorical ones. The ratio of the incidence of an aortic aneurysm or dissection in individuals with blood group A, AB or B compared to those with blood group O (the incidence rate ratio) was analyzed using log-linear Poisson regression models. The models included ABO blood group (A, AB, B or O) and were adjusted for age (expressed as natural cubic spline with 5 equally spaced knots), gender (female or male), and calendar year of observation (expressed as natural cubic spline with 5 equally spaced knots). In alternative analyses, ABO blood groups were instead

For peer review only - http://bmjopen.bmj.com/site/about/guidelines.xhtml

classified as O or non-O. Interactions between age and blood group and age and sex were also assessed by including interaction terms in the Poisson regression model.

All data processing and statistical analyses were performed with SAS statistical analysis software (version 9.4, SAS Institute, Inc., Cary, NC). All *p*-values below 0.05 were considered statistically significant. The creation of SCANDAT2 and the conduct of this study were approved by all regional ethics committees in Sweden, the Danish Scientific Ethics Committee and the Danish Data Protection Agency.

For peer review only

RESULTS

Study population and follow-up

The donor cohort consisted of 1 164 561 blood donors with a median follow-up time of 17.9 years, and the patient cohort included 961 637 recipients of blood transfusions with a median follow-up time of 8.9 years. Donors had a median age of 28 years at entry and were less often female (47.1%), whereas patients had a median age of 69 years, and were predominantly female (58.3%). The distribution of the different blood groups was similar in the two cohorts, with blood group O comprising about 38% of each cohort (Table 1).

Overall risk of aortic event

Altogether, there were 716 116 donors with a non-O blood group and 448 445 with blood group O, with a total follow-up time of 12 827 612 and 7 990 859 person-years, respectively. We observed 3 086 events (aortic aneurysm or dissection) in donors with non-O blood group compared to 1 977 events in donors with blood group O. The resulting incidence rate ratio (IRR) for any aortic event in donors with a non-O blood group compared to blood group O was 0.98 (95% confidence interval [CI], 0.93-1.04) (Table 2). The patient cohort included 593 040 patients with a non-O blood group and 368 597 patients with blood group O with a combined follow-up time of 5 266 210 and 3 305 968 person-years, respectively. Throughout follow-up, an aortic event (aortic aneurysm or dissection) was diagnosed in 9 567 patients with a non-O blood compared to 6 054 events in those with blood group O. The IRR for such events in patients with non-O blood was 1.00 (95% CI, 0.97-1.03) compared to those with blood group O.

No significant differences were observed when blood groups A, B, AB were compared with blood group O except for blood group B having an IRR 0.90 (95% CI, 0.85-0.96) for the occurrence of any aortic event in the patient cohort (Tables 2-4).

Risk of aortic dissection

Aortic dissection occurred in 587 non-O donors and 414 O donors during the study period,

For peer review only - http://bmjopen.bmj.com/site/about/guidelines.xhtml

For peer review only

corresponding to an IRR of 0.89 (95% CI, 0.78-1.01) for non-O donors compared to blood group O donors (Table 3). Aortic dissection was observed in 1 272 patients with non-O blood and 799 patients with blood group O, with an IRR of 1.01 (95% CI, 0.92-1.10). There were no differences in risk of aortic dissection in patients with blood groups A, B or AB compared to those with blood group O (Table 3).

Risk of aortic aneurysm

Aortic aneurysm was diagnosed in 2 781 donors with non-O blood and 1 740 O donors, resulting in an IRR of 1.00 (95% CI, 0.95-1.07) for non-O donors compared to O donors (Table 4). For the patient cohort, the corresponding numbers were 8 906 and 5 648, respectively, resulting in an IRR of 1.00 (95% CI, 0.96-1.03). Similar results were obtained in the analysis of the risk of aortic aneurysm in patients with blood groups A and AB. However, patients with blood group B had a significantly lower risk of experiencing an aortic event when compared to patients with blood group O with an IRR of 0.89 (95% CI, 0.84-0.94) (Table 2).

For peer review only

For peer review only - http://bmjopen.bmj.com/site/about/guidelines.xhtml

COMMENT

Here, we present results from the hitherto largest study of the association between ABO blood groups and aortic aneurysm or dissection with more than two million Swedish blood donors and transfusion recipients, a total of 20 684 aortic events and a combined follow-up time of almost 30 million person-years. The present study did not demonstrate a statistically significant association between ABO blood group and aortic disease with the exception of an inconsistent association in the patient cohort between blood group B and aortic aneurysm.

Previously, Fatic *et. al.* showed that a higher proportion of patients diagnosed with AAA had blood group O when compared to blood donors in the normal population (11), whereas Viklander *et. al.* could not find any association between blood groups and the risk of developing AAA or AAA rupture (10). It has been reported that non-O blood groups (*i.e.* blood groups A, B and AB) are associated with significantly higher risk of venous thromboembolism and cardiovascular disease, including myocardial infarction, peripheral vascular disease and cerebrovascular stroke (4-8). This could, in part, be explained by blood group O patients having 25-30% lower levels of circulating Factor VIII (FVIII) and von Willebrand factor (VWF) (15). However, Dentali *et. al.* demonstrated that non-O patients were at an increased risk of VTE even after adjustment for different levels of FVIII/VWF. Furthermore, it has been shown that that the genetic variation of ABO is associated with atherosclerosis and biomarkers of vascular inflammation (16), both of which are recognized risk factors for aortic aneurysm formation and aortic dissection (1, 17). The normal physiology of the vascular system relies on a negatively charged vascular wall, which repels the similarly negatively charged red blood cells and many negatively charged circulating proteins. A reduction of the negative charge may make the vascular wall more prone to thrombus formation and may also have an effect on the transportation of charged circulating proteases and cytokines into the vessel wall (18, 19). ABO blood groups and Rhesus antigen status have both been shown to influence the size of the charge potential (20), but the findings of the present study indicate that the previously described pathophysiological mechanisms of cardiovascular disease associated with ABO blood groups do not seem to translate to the formation of aortic aneurysms or the development of aortic dissection.

For peer review only - http://bmjopen.bmj.com/site/about/guidelines.xhtml

For peer review only

The differences in risk of cardiovascular disease between non-O and O individuals has previously been suggested as a specific effect of blood group A (21, 22). The analyses of different ABO blood groups in the present study could not demonstrate a specific effect of blood group A. Instead, our analyses showed a protective effect of blood group B on aortic disease in the patient cohort, primarily driven by significantly lower risk of aortic aneurysm development. To our knowledge, there are no previous reports suggesting an association between blood group B and cardiovascular disease and, thus, we have no plausible pathophysiological explanation for the specific effect of blood group B in this study. Furthermore, this association was not demonstrated in the donor cohort and, therefore, our speculation is that the protective effect of blood group B on the incidence of aortic disease might have been a chance finding.

The results presented in this study need some careful consideration. First, the donor and the patient cohort both represent selected populations and not the general population. However, to our knowledge, there are no data on blood group distributions in large cohorts of randomly selected individuals and therefore, donors or patients have generally served as control groups in similar studies in the past (4, 5, 6, 7, 8, 9, 12, 13). Donors represent a selected cohort of healthy individuals, whereas the patient cohort has a higher prevalence of co-morbidities compared to the general population. Despite the differences in health profile, this study did not demonstrate any major differences in blood group distribution between these two groups and therefore, we have no reason to believe that the distributions in donors and patients are not reflective of those in the general population. The external validity of the study might, therefore, be affected. Aortic aneurysm and dissection generally affect individuals older than the average age of blood donors. However, we did not find a significant interaction between ABO blood group and age. Furthermore, The SCANDAT2 database does not include patient genotyping and, therefore, we cannot fully assess the distribution of O alleles in blood group A and B patients. The use of hospital registers carries the inherent risk of misclassification, but it is unlikely that it relates to patient blood group. The limitations of the ICD diagnosis classification system did not permit us to definitively separate non-ruptured from ruptured aortic aneurysms and, thus, these were analyzed together, despite

For peer review only - http://bmjopen.bmj.com/site/about/guidelines.xhtml

ruptured aortic aneurysms potentially having larger similarities with aortic dissections. This should not, however, influence results from the analyses of the combination of aortic aneurysm and dissection.

On the other hand, there are several strengths to this study. It is by far the largest study investigating the association between ABO blood groups and aortic disease. The use of nationwide population and health registers allowed for complete follow-up and adds to the strength of the study. Blood group data was recorded prospectively and has high validity. Even though patients are potentially aware of their blood group, it is unlikely that such awareness has influenced their behavior. The prevalence of other risk factors for aortic disease should be similar between the different blood groups and, thereby, it should not have a confounding effect on the results of the analyses.

In conclusion, this large study did not demonstrate a significant association between ABO blood group and the risk of aortic disease. A possible protective effect of blood group B was, however observed in the patient cohort. Further studies are needed to confirm the presence and strength of this association.

DATA AVAILABILITY STATEMENT

No additional data available

FUNDING

The SCANDAT2 database was made possible through grants to Gustaf Edgren from the Swedish Research Council (2017-01954) and the Danish Research Council.

COMPETING INTERESTS STATEMENT

The authors have no conflicts of interest to declare.

AUTHOR STATEMENT

IZ contributed to the conception of the study and participated in designing and drafting the manuscript

GE and AM contributed to the conception of the study and were responsible for analysis and interpretation of data for the work;

SN participated in drafting the manuscript and contributed to the interpretation of the work

All authors agreed to the final approval of the version to be published

For peer review only - http://bmjopen.bmj.com/site/about/guidelines.xhtml

REFERENCES

1. Landenhed M, Engstrom G, Gottsater A, Caulfield MP, Hedblad B, Newton-Cheh C, et al. Risk profiles for aortic dissection and ruptured or surgically treated aneurysms: a prospective cohort study. J Am Heart Assoc. 2015;4(1):e001513.

2. Hagan PG, Nienaber CA, Isselbacher EM, Bruckman D, Karavite DJ, Russman PL, et al. The International Registry of Acute Aortic Dissection (IRAD): new insights into an old disease. JAMA. 2000;283(7):897-903.

3. Dahlof B. Cardiovascular disease risk factors: epidemiology and risk assessment. Am J Cardiol. 2010;105(1 Suppl):3A-9A.

4. Vasan SK, Rostgaard K, Majeed A, Ullum H, Titlestad KE, Pedersen OB, et al. ABO Blood Group and Risk of Thromboembolic and Arterial Disease: A Study of 1.5 Million Blood Donors. Circulation. 2016;133(15):1449-57; discussion 57.

5. Dentali F, Sironi AP, Ageno W, Crestani S, Franchini M. ABO blood group and vascular disease: an update. Semin Thromb Hemost. 2014;40(1):49-59.

6. Dentali F, Sironi AP, Ageno W, Turato S, Bonfanti C, Frattini F, et al. Non-O blood type is the commonest genetic risk factor for VTE: results from a meta-analysis of the literature. Semin Thromb Hemost. 2012;38(5):535-48.

7. Franchini M, Favaloro EJ, Targher G, Lippi G. ABO blood group, hypercoagulability, and cardiovascular and cancer risk. Crit Rev Clin Lab Sci. 2012;49(4):137-49.

8. Wu O, Bayoumi N, Vickers MA, Clark P. ABO(H) blood groups and vascular disease: a systematic review and meta-analysis. J Thromb Haemost. 2008;6(1):62-9.

9. Viklander G, Wallinder J, Henriksson AE. ABO blood groups and abdominal aortic aneurysm. Transfus Apher Sci. 2012;47(3):351-3.

10. Fatic N, Lukac H, Radojevic N, Simanic I, Banzic I, Pajovic B. O blood group as an indicator for abdominal aortic aneurysm. Eur Rev Med Pharmacol Sci. 2015;19(16):2997-3000.

11. Fatic N, Nikolic A, Vukmirovic M, Radojevic N, Zornic N, Banzic I, et al. Blood groups and acute aortic dissection type III. Arch Med Sci. 2017;13(3):597-600.

For peer review only - http://bmjopen.bmj.com/site/about/guidelines.xhtml

12. Edgren G, Hjalgrim H, Tran TN, Rostgaard K, Shanwell A, Titlestad K, et al. A population-based binational register for monitoring long-term outcome and possible disease concordance among blood donors and recipients. Vox sanguinis. 2006;91(4):316-23.

13. Edgren G, Rostgaard K, Vasan SK, Wikman A, Norda R, Pedersen OB, et al. The new Scandinavian Donations and Transfusions database (SCANDAT2): a blood safety resource with added versatility. Transfusion. 2015;55(7):1600-6.

14. Ludvigsson JF, Otterblad-Olausson P, Pettersson BU, Ekbom A. The Swedish personal identity number: possibilities and pitfalls in healthcare and medical research. Eur J Epidemiol. 2009;24(11):659-67.

15. Jenkins PV, O'Donnell JS. ABO blood group determines plasma von Willebrand factor levels: a biologic function after all? Transfusion. 2006;46(10):1836-44.

16. Larson NB, Bell EJ, Decker PA, Pike M, Wassel CL, Tsai MY, et al. ABO blood group associations with markers of endothelial dysfunction in the Multi-Ethnic Study of Atherosclerosis. Atherosclerosis. 2016;251:422-9.

17. Stone JR, Bruneval P, Angelini A, Bartoloni G, Basso C, Batoroeva L, et al. Consensus statement on surgical pathology of the aorta from the Society for Cardiovascular Pathology and the Association for European Cardiovascular Pathology: I. Inflammatory diseases. Cardiovasc Pathol. 2015;24(5):267-78.

18. Srinivasan S, Sawyer PN. Role of surface charge of the blood vessel wall, blood cells, and prosthetic materials in intravascular thrombosis. J Colloid Interface Sci. 1970;32(3):456-63.

19. Michel JB, Jondeau G, Milewicz DM. From genetics to response to injury: vascular smooth muscle cells in aneurysms and dissections of the ascending aorta. Cardiovasc Res. 2018;114(4):578-89.

20. Thubrikar MJ, Cadoff I, Youdin M, Reich T. Effect of blood types and vessel wall surface charge on thrombogenicity: preliminary report. Bull N Y Acad Med. 1975;51(8):974-83.

21. Medalie JH, Levene C, Papier C, Goldbourt U, Dreyfuss F, Oron D, et al. Blood groups, myocardial infarction and angina pectoris among 10,000 adult males. N Engl J Med. 1971;285(24):1348-53.

For peer review only - http://bmjopen.bmj.com/site/about/guidelines.xhtml

22. Platt D, Muhlberg W, Kiehl L, Schmitt-Ruth R. ABO blood group system, age, sex, risk factors and cardiac infarction. Arch Gerontol Geriat. 1985;4(3):241-9.

For peer review only

Table 1. Characteristics of study participants.

	Donor cohort		Patient cohort	
Number of subjects	1 164 561		961 637	
Female sex, N (%)	548 234	(47.1)	560 537	(58.3)
Age at start of follow-up, N (%)				
18-40 years	910 537	(78.2)	134 160	(14.0)
41-60 years	246 772	(21.2)	185 080	(19.2)
61-75 years	7 252	(0.6)	307 216	(31.9)
>75 years	0	(0.0)	335 181	(34.9)
Median (IQR)*	28	(22-38)	69	(54-79)
ABO blood group, N (%)				
A	522 364	(44.9)	444 075	(46.2)
AB	62 001	(5.3)	47 610	(5.0)
B	131 751	(11.3)	101 355	(10.5)
O	448 445	(38.5)	368 597	(38.3)
Born in Sweden, N (%)	1 076 286	(92.4)	858 098	(89.2)

*SD denotes standard deviation; IQR denotes interquartile range.

For peer review only

Table 2. Relative risk of aortic dissection or aortic aneurysm in relation to ABO blood group, presented separately for the donor and recipient cohorts.

	**Donor cohort**		**Patient cohort**	
ABO blood group	Events/ person-years	Incidence rate ratio (95% confidence interval)	Events/ person-years	Incidence rate ratio (95% confidence interval)
A	2 281/9 344 274	1.00 (0.94-1.06)	7 343/3 911 097	1.02 (0.98-1.05)
AB	268/1 125 603	0.94 (0.83-1.07)	783/ 426 761	1.04 (0.96-1.12)
B	537/2 348 996	0.93 (0.85-1.03)	1 441/ 907 053	0.90 (0.85-0.96)
O	1 977/7 984 924	1.00 (ref)	6 054/3 292 052	1.00 (ref)
Non-O	3 086/12 818 872	0.98 (0.93-1.04)	9 567/5 244 912	1.00 (0.97-1.03)
O	1 977/7 984 924	1.00 (ref)	6 054/3 292 052	1.00 (ref)

For peer review only

For peer review only - http://bmjopen.bmj.com/site/about/guidelines.xhtml

Table 3. Relative risk of aortic dissection in relation to ABO blood group, presented separately for the donor and recipient cohorts.

	Donor cohort		Patient cohort	
ABO blood group	Events/ person-years	Incidence rate ratio (95% confidence interval)	Events/ person-years	Incidence rate ratio (95% confidence interval)
A	426/9 350 727	0.89 (0.77-1.01)	933/3 927 796	0.98 (0.89-1.08)
AB	55/1 126 318	0.93 (0.70-1.23)	116/ 428 385	1.16 (0.95-1.41)
B	106/2 350 567	0.88 (0.71-1.09)	223/ 910 029	1.05 (0.91-1.22)
O	414/7 990 859	1.00 (ref)	799/3 305 968	1.00 (ref)
Non-O	587/12 827 612	0.89 (0.78-1.01)	1 272/5 266 210	1.01 (0.92-1.10)
O	414/7 990 859	1.00 (ref)	799/3 305 968	1.00 (ref)

For peer review only

For peer review only - http://bmjopen.bmj.com/site/about/guidelines.xhtml

Table 4. Relative risk of aortic aneurysm in relation to ABO blood group, presented separately for the donor and recipient cohorts.

ABO blood group	Donor cohort		Patient cohort	
	Events/ person-years	Incidence rate ratio (95% confidence interval)	Events/ person-years	Incidence rate ratio (95% confidence interval)
A	2 056/9 345 277	1.02 (0.96-1.09)	6 852/3 912 179	1.02 (0.98-1.05)
AB	242/1 125 746	0.97 (0.85-1.11)	733/ 426 928	1.04 (0.97-1.13)
B	483/2 349 202	0.95 (0.86-1.05)	1 321/ 907 322	0.89 (0.84-0.94)
O	1 740/7 985 856	1.00 (ref)	5 648/3 292 796	1.00 (ref)
Non-O	2 781/12 820 224	1.00 (0.95-1.07)	8 906/5 246 429	1.00 (0.96-1.03)
O	1 740/7 985 856	1.00 (ref)	5 648/3 292 796	1.00 (ref)

For peer review only

For peer review only - http://bmjopen.bmj.com/site/about/guidelines.xhtml

11

For peer review only

Supplementary table 1. ICD codes used for group classifications

ICD version	Group	Diagnosis	Description
10	1	I710	Aortic dissection
10	1	I711	Thoracic aortic aneurysm, ruptured
10	1	I713	Abdominal aortic aneurysm, ruptured
10	1	I715	Thoracoabdominal aortic aneurysm, ruptured
10	1	I718	Aortic aneurysm with unspecified location, ruptured
10	1	I712	Thoracic aortic aneurysm, non-ruptured
10	1	I714	Abdominal aortic aneurysm, non-ruptured
10	1	I716	Thoracoabdominal aortic aneurysm, non-ruptured
10	1	I719	Aortic aneurysm with unspecified location, non-ruptured
10	2	I710	Aortic dissection
10	3	I711	Thoracic aortic aneurysm, ruptured
10	3	I713	Abdominal aortic aneurysm, ruptured
10	3	I715	Thoracoabdominal aortic aneurysm, ruptured
10	3	I718	Aortic aneurysm with unspecified location, ruptured
10	3	I712	Thoracic aortic aneurysm, non-ruptured
10	3	I714	Abdominal aortic aneurysm, non-ruptured
10	3	I716	Thoracoabdominal aortic aneurysm, non-ruptured
10	3	I719	Aortic aneurysm with unspecified location, non-ruptured
9	1	4410	Aortic dissection
9	1	4411	Thoracic aortic aneurysm, ruptured
9	1	4413	Abdominal aortic aneurysm, ruptured
9	1	4416	Thoracoabdominal aortic aneurysm, ruptured
9	1	4415	Aortic aneurysm with unspecified location, ruptured
9	1	4412	Thoracic aortic aneurysm, non-ruptured
9	1	4414	Abdominal aortic aneurysm, non-ruptured
9	1	4417	Thoracoabdominal aortic aneurysm, non-ruptured
9	1	4419	Aortic aneurysm with unspecified location, non-ruptured
9	2	4410	Aortic dissection
9	3	4411	Thoracic aortic aneurysm, ruptured
9	3	4413	Abdominal aortic aneurysm, ruptured
9	3	4416	Thoracoabdominal aortic aneurysm, ruptured
9	3	4415	Aortic aneurysm with unspecified location, ruptured
9	3	4412	Thoracic aortic aneurysm, non-ruptured
9	3	4414	Abdominal aortic aneurysm, non-ruptured
9	3	4417	Thoracoabdominal aortic aneurysm, non-ruptured
9	3	4419	Aortic aneurysm with unspecified location, non-ruptured
8	1	4410	Aortic dissection
8	1	4411	Thoracic aortic aneurysm
8	1	4412	Abdominal aortic aneurysm
8	1	4419	Aortic aneurysm with unspecified location, non-ruptured
8	2	4410	Aortic dissection
8	3	4411	Thoracic aortic aneurysm
8	3	4412	Abdominal aortic aneurysm
8	3	4419	Aortic aneurysm with unspecified location, non-ruptured
7	1	45100	Aortic dissection
7	1	45110	Aortic dissection

For peer review only

Supplementary table 2. Group description

Group	Group name	Description
1	Aortic disease	Aortic aneurysm and aortic dissection
2	Aortic dissection	Aortic dissection
3	Aortic aneurysm	Non-ruptured or ruptured aortic aneurysm

For peer review only

STROBE Statement—Checklist of items that should be included in reports of ***case-control studies***

	Item No	Recommendation
**Title and abstract**	1	(*a*) Indicate the study's design with a commonly used term in the title or the abstract
		(*b*) Provide in the abstract an informative and balanced summary of what was done and what was found
**Introduction**		
Background/rationale	2	Explain the scientific background and rationale for the investigation being reported
Objectives	3	State specific objectives, including any prespecified hypotheses
**Methods**		
Study design	4	Present key elements of study design early in the paper
Setting	5	Describe the setting, locations, and relevant dates, including periods of recruitment, exposure, follow-up, and data collection
Participants	6	(*a*) Give the eligibility criteria, and the sources and methods of case ascertainment and control selection. Give the rationale for the choice of cases and controls
		(*b*) For matched studies, give matching criteria and the number of controls per case
Variables	7	Clearly define all outcomes, exposures, predictors, potential confounders, and effect modifiers. Give diagnostic criteria, if applicable
Data sources/ measurement	8*	For each variable of interest, give sources of data and details of methods of assessment (measurement). Describe comparability of assessment methods if there is more than one group
Bias	9	Describe any efforts to address potential sources of bias
Study size	10	Explain how the study size was arrived at
Quantitative variables	11	Explain how quantitative variables were handled in the analyses. If applicable, describe which groupings were chosen and why
Statistical methods	12	(*a*) Describe all statistical methods, including those used to control for confounding
		(*b*) Describe any methods used to examine subgroups and interactions
		(*c*) Explain how missing data were addressed
		(*d*) If applicable, explain how matching of cases and controls was addressed
		(*e*) Describe any sensitivity analyses
**Results**		
Participants	13*	(a) Report numbers of individuals at each stage of study—eg numbers potentially eligible, examined for eligibility, confirmed eligible, included in the study, completing follow-up, and analysed
		(b) Give reasons for non-participation at each stage
		(c) Consider use of a flow diagram
Descriptive data	14*	(a) Give characteristics of study participants (eg demographic, clinical, social) and information on exposures and potential confounders
		(b) Indicate number of participants with missing data for each variable of interest
Outcome data	15*	Report numbers in each exposure category, or summary measures of exposure
Main results	16	(*a*) Give unadjusted estimates and, if applicable, confounder-adjusted estimates and their precision (eg, 95% confidence interval). Make clear which confounders were adjusted for and why they were included
		(*b*) Report category boundaries when continuous variables were categorized
		(*c*) If relevant, consider translating estimates of relative risk into absolute risk for a meaningful time period

For peer review only - http://bmjopen.bmj.com/site/about/guidelines.xhtml

Other analyses	17	Report other analyses done—eg analyses of subgroups and interactions, and sensitivity analyses
**Discussion**		
Key results	18	Summarise key results with reference to study objectives
Limitations	19	Discuss limitations of the study, taking into account sources of potential bias or imprecision. Discuss both direction and magnitude of any potential bias
Interpretation	20	Give a cautious overall interpretation of results considering objectives, limitations, multiplicity of analyses, results from similar studies, and other relevant evidence
Generalisability	21	Discuss the generalisability (external validity) of the study results
**Other information**		
Funding	22	Give the source of funding and the role of the funders for the present study and, if applicable, for the original study on which the present article is based

*Give information separately for cases and controls.

Note: An Explanation and Elaboration article discusses each checklist item and gives methodological background and published examples of transparent reporting. The STROBE checklist is best used in conjunction with this article (freely available on the Web sites of PLoS Medicine at http://www.plosmedicine.org/, Annals of Internal Medicine at http://www.annals.org/, and Epidemiology at http://www.epidem.com/). Information on the STROBE Initiative is available at http://www.strobe-statement.org.

For peer review only

For peer review only - http://bmjopen.bmj.com/site/about/guidelines.xhtml

ABO blood group and the risk of aortic disease - A nationwide cohort study

Journal:	*BMJ Open*
Manuscript ID	bmjopen-2019-036040.R1
Article Type:	Original research
Date Submitted by the Author:	11-May-2020
Complete List of Authors:	Zindovic, Igor; Department of Cardiothoracic Surgery, Clinical Sciences, Lund University, Skane University Hospital<br>Edgren, Gustaf; Karolinska Universitetssjukhuset, Department of Medicine, Solna, Clinical Epidemiology Division, Karolinska Institutet, Stockholm, Sweden; Department of Cardiology, Södersjukhuset, Stockholm, Sweden<br>Nozohoor, Shahab; Department of Cardiothoracic Surgery, Clinical Sciences, Lund University, Skane University Hospital<br>Majeed, Ammar; Department of Medicine, Solna, Clinical Epidemiology Division, Karolinska Institutet, Stockholm, Sweden; Department of Gastroenterology, The Alfred Health, Melbourne, Victoria, Australia
**Primary Subject Heading**:	Epidemiology
Secondary Subject Heading:	Cardiovascular medicine
Keywords:	EPIDEMIOLOGY, Thoracic surgery < SURGERY, Bleeding disorders & coagulopathies < HAEMATOLOGY

I, the Submitting Author has the right to grant and does grant on behalf of all authors of the Work (as defined in the below author licence), an exclusive licence and/or a non-exclusive licence for contributions from authors who are: i) UK Crown employees; ii) where BMJ has agreed a CC-BY licence shall apply, and/or iii) in accordance with the terms applicable for US Federal Government officers or employees acting as part of their official duties; on a worldwide, perpetual, irrevocable, royalty-free basis to BMJ Publishing Group Ltd ("BMJ") its licensees and where the relevant Journal is co-owned by BMJ to the co-owners of the Journal, to publish the Work in this journal and any other BMJ products and to exploit all rights, as set out in our licence.

The Submitting Author accepts and understands that any supply made under these terms is made by BMJ to the Submitting Author unless you are acting as an employee on behalf of your employer or a postgraduate student of an affiliated institution which is paying any applicable article publishing charge ("APC") for Open Access articles. Where the Submitting Author wishes to make the Work available on an Open Access basis (and intends to pay the relevant APC), the terms of reuse of such Open Access shall be governed by a Creative Commons licence – details of these licences and which Creative Commons licence will apply to this Work are set out in our licence referred to above.

Other than as permitted in any relevant BMJ Author's Self Archiving Policies, I confirm this Work has not been accepted for publication elsewhere, is not being considered for publication elsewhere and does not duplicate material already published. I confirm all authors consent to publication of this Work and authorise the granting of this licence.

For peer review only

ABO blood group and the risk of aortic disease - A nationwide cohort study

Igor Zindovic MD[1], Gustaf Edgren MD PhD[2,3], Shahab Nozohoor MD PhD[1], Ammar Majeed MD PhD[2,4,5]

[1]Lund University, Skåne University Hospital, Department of Clinical Sciences, Department of Cardiothoracic Surgery, Lund, Sweden; [2]Department of Medicine, Solna, Clinical Epidemiology Division, Karolinska Institutet, Stockholm, Sweden; [3]Department of Cardiology, Södersjukhuset, Stockholm, Sweden; [4]Department of Gastroenterology, The Alfred Health, Melbourne, Victoria, Australia; [5]Central Clinical School, Monash University, Melbourne, Victoria, Australia.

Corresponding author: Igor Zindovic, MD

Department of Cardiothoracic Surgery

Skåne University Hospital, SE-221 85 Lund, Sweden

Phone: +46 46-171000; E-mail: igor.zindovic@med.lu.se

Co-author contact information:

Gustaf.Edgren@ki.se; Shahab.Nozohoor@med.lu.se; Ammar.Majeed@ki.se

For peer review only

ABSTRACT

Objectives: To analyze the association between ABO blood group and aortic disease using data on blood donors and transfused patients from Sweden.

Design: This was a retrospective study using data from the Swedish portion of the Scandinavian Donations and Transfusions database (SCANDAT2). The association between ABO blood group and aortic disease was analyzed using log-linear Poisson regression models and presented as incidence rate ratios (IRR:s).

Setting: Swedish population-based study.

Participants: The study cohort consisted of 1 164 561 Swedish blood donors and 961 637 transfused patients with a combined follow-up time of 29 390 649 person-years.

Primary and secondary outcome measures: IRR:s of aortic events (*i.e.* aortic aneurysms and/or aortic dissections) in relation to patient blood group.

Results: A total of 20 684 aortic events occurred during the study period. Non-O donors and patients had similar incidence of aortic events when compared to blood group O donors and patients with an IRR of 0.98 (95% confidence interval [CI], 0.93-1.04) and 1.00 (95% CI, 0.97-1.03), respectively. There were no differences between non-O and blood group O individuals when aortic dissections and aortic aneurysms were analyzed separately. Blood group B conferred a lower risk of aortic aneurysms in the patient cohort when compared to blood group O (IRR, 0.90; 95% CI, 0.85-0.96).

Conclusions: In the present study, there were no statistically significant associations between ABO blood group and the risk of aortic disease. A possible protective effect of blood group B was observed in the patient cohort but this finding requires further investigation.

For peer review only

For peer review only - http://bmjopen.bmj.com/site/about/guidelines.xhtml

STRENGTHS AND LIMITATIONS OF THE STUDY

- Data were collected from a quality registry covering all blood donations and transfusions 1968 to 2012.
- This study is strengthened by complete follow-up of the study cohort.
- This is by far the largest study assessing the association between blood groups and aortic disease.
- The study is limited by donors representing a selected cohort of healthy individuals, whereas the patient cohort has a higher prevalence of co-morbidities compared to the general population.
- Another limitation is that detailed information on co-morbidities of individuals in the study population was not known.

FUNDING

The SCANDAT2 database was made possible through grants to Gustaf Edgren from the Swedish Research Council (2017-01954) and the Danish Research Council.

COMPETING INTERESTS STATEMENT

The authors have no conflicts of interest to declare.

DATA AVAILABILITY:

No additional data available.

For peer review only

INTRODUCTION

Aortic aneurysm and dissection are potentially life-threatening conditions that share common risk factors—age, hypertension, atherosclerosis, and smoking—with other types of cardiovascular disease (1-3). The association between ABO blood groups and cardiovascular disease and venous thromboembolism (VTE) has been widely reported, but the pathophysiological mechanisms are not fully understood (4-8).

Few studies have examined the association between ABO blood group and aortic disease (9-11). In an analysis of 504 patients with abdominal aortic aneurysm (AAA), Viklander *et. al.* did not find a significant difference in distribution of blood groups between patients with AAA or ruptured AAA compared to a population-based control group (9). Fatic *et. al.* on the other hand, demonstrated that a significantly higher proportion of AAA patients had blood group O compared to a control group consisting of blood donors from the general population of Montenegro (10). Available research is limited by the small size of the studies, conflicting results and the omission of patients with aortic dissection, warranting further examination of the relationship between blood groups and aortic disease.

The aim of the present study was to evaluate the association between ABO blood group and aortic disease using data from a large cohort of Swedish individuals derived from the Scandinavian Donations and Transfusions (SCANDAT2) database (12).

For peer review only

For peer review only - http://bmjopen.bmj.com/site/about/guidelines.xhtml

MATERIAL AND METHODS

Data source

The study was based on the Scandinavian Donations and Transfusions database (SCANDAT2). This is a computerized, combined donation and transfusion register using data on more than 1.6 million blood donors and 2 million recipients of blood products in Sweden from 1968 to 2012 and in Denmark from 1981 to 2012 (12, 13). Using the unique personal identity numbers assigned to all residents of the two countries, SCANDAT2 is linked to national population, death, and migration registers, allowing for the complete follow-up of all individuals in the register up to and including December 31, 2012 (14). The database is also linked to the national inpatient, outpatient, cause of death and cancer registries with information on registered diagnoses coded as the International Classification of Diseases (ICD) codes, versions 6-10.

Study Design

This was a retrospective data analysis based on individuals included in the SCANDAT2 register. For the purpose of these analyses, we only used data from the Swedish portion of the SCANDAT2 database and restricted the analysis to donors and patients who donated or received their first blood product after January 1, 1970, when the patient register in Sweden had good national coverage. Donors and recipients with a documented blood group in SCANDAT2, who had given or received at least one blood product between January 1, 1970 and December 31, 2012, were included in the study. We used a set of ICD codes to identify donors and recipients who were diagnosed with either aortic aneurysm or aortic dissection (Supplementary Table 1) based on information from the patient register. The different ICD codes were grouped into 2 different disease groups based on etiological factors or disease characteristics (Supplementary Table 2). Recognizing that aortic aneurysm and dissection share common risk factors and disease characteristics, we created a third group that encompassed all ICD codes for aortic aneurysm and dissection. Donors and recipients who had a diagnosis of aortic aneurysm and/or dissection prior to their first donation or transfusion were excluded from the study. In the present study, we used

For peer review only

the term "aortic disease" to reflect either aortic aneurysm or aortic dissection and the diagnosis of an aortic aneurysm or aortic dissection as an "aortic event".

Statistical analysis

Recognizing differences in demographics and comorbidities, we created separate cohorts for the donors and the recipients. In the analysis for the donor cohort, we followed the donors from the date of first electronically recorded blood donation in SCANDAT2 or the study start date (January 1, 1970), whichever came last. For the transfusion recipient cohort, individuals were followed from the start of the study (January 1, 1970) or 365 days after their first electronically recorded blood transfusion in SCANDAT2, whichever came last. We delayed the patients' entry into the study by one year after their first transfusion to reduce the risk of reverse causation bias (*i.e.* when the indication for their first transfusion would somehow be related to the subsequent occurrence of an aortic event). All study participants were followed up until either the date of diagnosis of aortic aneurysm or dissection, emigration, death or end of follow-up (December 31, 2012), whichever came first. As an individual could be diagnosed with more than one type of aortic aneurysm or have an aneurysm and dissection diagnosed at different time points during their lifetime, we allowed for the same individual to contribute time at risk and events into more than one disease group based on their diagnoses.

Baseline characteristics are presented as means and standard deviations for continuous variables, and frequencies and percentages for categorical ones. The ratio of the incidence of an aortic aneurysm or dissection in individuals with blood group A, AB or B compared to those with blood group O (the incidence rate ratio) was analyzed using log-linear Poisson regression models. The models included

For peer review only - http://bmjopen.bmj.com/site/about/guidelines.xhtml

ABO blood group (A, AB, B or O) and were adjusted for age (expressed as natural cubic spline with 5 equally spaced knots), gender (female or male), and calendar year of observation (expressed as natural cubic spline with 5 equally spaced knots). In alternative analyses, ABO blood groups were instead classified as O or non-O. Interactions between age and blood group and age and sex were also assessed by including interaction terms in the Poisson regression model.

All data processing and statistical analyses were performed with SAS statistical analysis software (version 9.4, SAS Institute, Inc., Cary, NC). All *p*-values below 0.05 were considered statistically significant. The creation of SCANDAT2 and the conduct of this study were approved by the Swedish Ethical Review Authority, the Danish Scientific Ethics Committee and the Danish Data Protection Agency.

Patient and Public Involvement

Apart from contributing with their personal study data, patients were not involved in this study.

For peer review only

RESULTS

Study population and follow-up

The donor cohort consisted of 1 164 561 blood donors with a median follow-up time of 17.9 years, and the patient cohort included 961 637 recipients of blood transfusions with a median follow-up time of 8.9 years. Donors had a median age of 28 years at entry and were less often female (47.1%), whereas patients had a median age of 69 years, and were predominantly female (58.3%). The distribution of the different blood groups was similar in the two cohorts, with blood group O comprising about 38% of each cohort (Table 1).

Overall risk of aortic event

Altogether, there were 716 116 donors with a non-O blood group and 448 445 with blood group O, with a total follow-up time of 12 827 612 and 7 990 859 person-years, respectively. We observed 3 086 events (aortic aneurysm or dissection) in donors with non-O blood group compared to 1 977 events in donors with blood group O. The resulting incidence rate ratio (IRR) for any aortic event in donors with a non-O blood group compared to blood group O was 0.98 (95% confidence interval [CI], 0.93-1.04) (Table 2). The patient cohort included 593 040 patients with a non-O blood group and 368 597 patients with blood group O with a combined follow-up time of 5 266 210 and 3 305 968 person-years, respectively. Throughout follow-up, an aortic event (aortic aneurysm or dissection) was diagnosed in 9 567 patients with a non-O blood compared to 6 054 events in those with blood group O. The IRR for such events in patients with non-O blood was 1.00 (95% CI, 0.97-1.03) compared to those with blood group O.

No significant differences were observed when blood groups A, B, AB were compared with blood group O except for blood group B having an IRR 0.90 (95% CI, 0.85-0.96) for the occurrence of any aortic event in the patient cohort (Tables 2-4).

Risk of aortic dissection

Aortic dissection occurred in 587 non-O donors and 414 O donors during the study period,

For peer review only - http://bmjopen.bmj.com/site/about/guidelines.xhtml
For peer review only

corresponding to an IRR of 0.89 (95% CI, 0.78-1.01) for non-O donors compared to blood group O donors (Table 3). Aortic dissection was observed in 1 272 patients with non-O blood and 799 patients with blood group O, with an IRR of 1.01 (95% CI, 0.92-1.10). There were no differences in risk of aortic dissection in patients with blood groups A, B or AB compared to those with blood group O (Table 3).

Risk of aortic aneurysm

Aortic aneurysm was diagnosed in 2 781 donors with non-O blood and 1 740 O donors, resulting in an IRR of 1.00 (95% CI, 0.95-1.07) for non-O donors compared to O donors (Table 4). For the patient cohort, the corresponding numbers were 8 906 and 5 648, respectively, resulting in an IRR of 1.00 (95% CI, 0.96-1.03). Similar results were obtained in the analysis of the risk of aortic aneurysm in patients with blood groups A and AB. However, patients with blood group B had a significantly lower risk of experiencing an aortic event when compared to patients with blood group O with an IRR of 0.89 (95% CI, 0.84-0.94) (Table 2).

For peer review only

For peer review only - http://bmjopen.bmj.com/site/about/guidelines.xhtml

DISCUSSION

Here, we present results from the hitherto largest study of the association between ABO blood groups and aortic aneurysm or dissection with more than two million Swedish blood donors and transfusion recipients, a total of 20 684 aortic events and a combined follow-up time of almost 30 million person-years. The present study did not demonstrate a statistically significant association between ABO blood group and aortic disease with the exception of an inconsistent association in the patient cohort between blood group B and aortic aneurysm.

Previously, Fatic *et. al.* showed that a higher proportion of patients diagnosed with AAA had blood group O when compared to blood donors in the normal population (11), whereas Viklander *et. al.* could not find any association between blood groups and the risk of developing AAA or AAA rupture (10). It has been reported that non-O blood groups (*i.e.* blood groups A, B and AB) are associated with significantly higher risk of venous thromboembolism and cardiovascular disease, including myocardial infarction, peripheral vascular disease and cerebrovascular stroke (4-8). This could, in part, be explained by blood group O patients having 25-30% lower levels of circulating Factor VIII (FVIII) and von Willebrand factor (VWF) (15). However, Dentali *et. al.* demonstrated that non-O patients were at an increased risk of VTE even after adjustment for different levels of FVIII/VWF. Furthermore, it has been shown that that the genetic variation of ABO is associated with atherosclerosis and biomarkers of vascular inflammation (16), both of which are recognized risk factors for aortic aneurysm formation and aortic dissection (1, 17). The normal physiology of the vascular system relies on a negatively charged vascular wall, which repels the similarly negatively charged red blood cells and many negatively charged circulating proteins. A reduction of the negative charge may make the vascular wall more prone to thrombus formation and may also have an effect on the transportation of charged circulating proteases and cytokines into the vessel wall (18, 19). ABO blood groups and Rhesus antigen status have both been shown to influence the size of the charge potential (20), but the findings of the present study indicate that the previously described pathophysiological mechanisms of cardiovascular disease associated with ABO blood groups do not seem to translate to the formation of aortic aneurysms or the development of aortic dissection.

For peer review only - http://bmjopen.bmj.com/site/about/guidelines.xhtml

The differences in risk of cardiovascular disease between non-O and O individuals has previously been suggested as a specific effect of blood group A (21, 22). The analyses of different ABO blood groups in the present study could not demonstrate a specific effect of blood group A. Instead, our analyses showed a protective effect of blood group B on aortic disease in the patient cohort, primarily driven by significantly lower risk of aortic aneurysm development. To our knowledge, there are no previous reports suggesting an association between blood group B and cardiovascular disease and, thus, we have no plausible pathophysiological explanation for the specific effect of blood group B in this study. Furthermore, this association was not demonstrated in the donor cohort and, therefore, our speculation is that the protective effect of blood group B on the incidence of aortic disease might have been a chance finding.

The results presented in this study need some careful consideration. First, the donor and the patient cohort both represent selected populations and not the general population. However, to our knowledge, there are no data on blood group distributions in large cohorts of randomly selected individuals and therefore, donors or patients have generally served as control groups in similar studies in the past (4, 5, 6, 7, 8, 9, 12, 13). Donors represent a selected cohort of healthy individuals, whereas the patient cohort has a higher prevalence of co-morbidities compared to the general population. Despite the differences in health profile, this study did not demonstrate any major differences in blood group distribution between these two groups and therefore, we have no reason to believe that the distributions in donors and patients are not reflective of those in the general population. The external validity of the study might, therefore, be affected. Aortic aneurysm and dissection generally affect individuals older than the average age of blood donors. However, we did not find a significant interaction between ABO blood group and age. Furthermore, The SCANDAT2 database does not include patient genotyping and, therefore, we cannot fully assess the distribution of O alleles in blood group A and B patients. The use of hospital registers carries the inherent risk of misclassification, but it is unlikely that it relates to patient blood group. The limitations of the ICD diagnosis classification system did not permit us to definitively separate non-ruptured from ruptured aortic aneurysms and, thus, these were analyzed together, despite

For peer review only - http://bmjopen.bmj.com/site/about/guidelines.xhtml

ruptured aortic aneurysms potentially having larger similarities with aortic dissections. This should not, however, influence results from the analyses of the combination of aortic aneurysm and dissection.

On the other hand, there are several strengths to this study. It is by far the largest study investigating the association between ABO blood groups and aortic disease. The use of nationwide population and health registers allowed for complete follow-up and adds to the strength of the study. Blood group data was recorded prospectively and has high validity. Even though patients are potentially aware of their blood group, it is unlikely that such awareness has influenced their behavior. The prevalence of other risk factors for aortic disease should be similar between the different blood groups and, thereby, it should not have a confounding effect on the results of the analyses.

In conclusion, this large study did not demonstrate a significant association between ABO blood group and the risk of aortic disease. A possible protective effect of blood group B was, however observed in the patient cohort. Further studies are needed to confirm the presence and strength of this association.

Authorship statement

The conception and design of the study was performed by Igor Zindovic, Gustaf Edgren and Ammar Majeed. Gustaf Edgren and Ammar Majeed performed the statistical analyses and Igor Zindovic and Shahab Nozohoor produced the first draft of the manuscript. The data was interpreted by all co-authors. The manuscript was critically reviewed for important intellectual content and the final version was approved by all authors. All authors had full access to all the data (including statistical reports and tables) of the study and can take responsibility for the integrity of the data and the accuracy of the data analysis.

WORD COUNT

3564

For peer review only - http://bmjopen.bmj.com/site/about/guidelines.xhtml

REFERENCES

1. Landenhed M, Engstrom G, Gottsater A, Caulfield MP, Hedblad B, Newton-Cheh C, et al. Risk profiles for aortic dissection and ruptured or surgically treated aneurysms: a prospective cohort study. J Am Heart Assoc. 2015;4(1):e001513.

2. Hagan PG, Nienaber CA, Isselbacher EM, Bruckman D, Karavite DJ, Russman PL, et al. The International Registry of Acute Aortic Dissection (IRAD): new insights into an old disease. JAMA. 2000;283(7):897-903.

3. Dahlof B. Cardiovascular disease risk factors: epidemiology and risk assessment. Am J Cardiol. 2010;105(1 Suppl):3A-9A.

4. Vasan SK, Rostgaard K, Majeed A, Ullum H, Titlestad KE, Pedersen OB, et al. ABO Blood Group and Risk of Thromboembolic and Arterial Disease: A Study of 1.5 Million Blood Donors. Circulation. 2016;133(15):1449-57; discussion 57.

5. Dentali F, Sironi AP, Ageno W, Crestani S, Franchini M. ABO blood group and vascular disease: an update. Semin Thromb Hemost. 2014;40(1):49-59.

6. Dentali F, Sironi AP, Ageno W, Turato S, Bonfanti C, Frattini F, et al. Non-O blood type is the commonest genetic risk factor for VTE: results from a meta-analysis of the literature. Semin Thromb Hemost. 2012;38(5):535-48.

7. Franchini M, Favaloro EJ, Targher G, Lippi G. ABO blood group, hypercoagulability, and cardiovascular and cancer risk. Crit Rev Clin Lab Sci. 2012;49(4):137-49.

8. Wu O, Bayoumi N, Vickers MA, Clark P. ABO(H) blood groups and vascular disease: a systematic review and meta-analysis. J Thromb Haemost. 2008;6(1):62-9.

9. Viklander G, Wallinder J, Henriksson AE. ABO blood groups and abdominal aortic aneurysm. Transfus Apher Sci. 2012;47(3):351-3.

10. Fatic N, Lukac H, Radojevic N, Simanic I, Banzic I, Pajovic B. O blood group as an indicator for abdominal aortic aneurysm. Eur Rev Med Pharmacol Sci. 2015;19(16):2997-3000.

11. Fatic N, Nikolic A, Vukmirovic M, Radojevic N, Zornic N, Banzic I, et al. Blood groups and acute aortic dissection type III. Arch Med Sci. 2017;13(3):597-600.

For peer review only

For peer review only - http://bmjopen.bmj.com/site/about/guidelines.xhtml

12. Edgren G, Rostgaard K, Vasan SK, Wikman A, Norda R, Pedersen OB, et al. The new Scandinavian Donations and Transfusions database (SCANDAT2): a blood safety resource with added versatility. Transfusion. 2015;55(7):1600-6.

13. Edgren G, Hjalgrim H, Tran TN, Rostgaard K, Shanwell A, Titlestad K, et al. A population-based binational register for monitoring long-term outcome and possible disease concordance among blood donors and recipients. Vox sanguinis. 2006;91(4):316-23.

14. Ludvigsson JF, Otterblad-Olausson P, Pettersson BU, Ekbom A. The Swedish personal identity number: possibilities and pitfalls in healthcare and medical research. Eur J Epidemiol. 2009;24(11):659-67.

15. Jenkins PV, O'Donnell JS. ABO blood group determines plasma von Willebrand factor levels: a biologic function after all? Transfusion. 2006;46(10):1836-44.

16. Larson NB, Bell EJ, Decker PA, Pike M, Wassel CL, Tsai MY, et al. ABO blood group associations with markers of endothelial dysfunction in the Multi-Ethnic Study of Atherosclerosis. Atherosclerosis. 2016;251:422-9.

17. Stone JR, Bruneval P, Angelini A, Bartoloni G, Basso C, Batoroeva L, et al. Consensus statement on surgical pathology of the aorta from the Society for Cardiovascular Pathology and the Association for European Cardiovascular Pathology: I. Inflammatory diseases. Cardiovasc Pathol. 2015;24(5):267-78.

18. Srinivasan S, Sawyer PN. Role of surface charge of the blood vessel wall, blood cells, and prosthetic materials in intravascular thrombosis. J Colloid Interface Sci. 1970;32(3):456-63.

19. Michel JB, Jondeau G, Milewicz DM. From genetics to response to injury: vascular smooth muscle cells in aneurysms and dissections of the ascending aorta. Cardiovasc Res. 2018;114(4):578-89.

20. Thubrikar MJ, Cadoff I, Youdin M, Reich T. Effect of blood types and vessel wall surface charge on thrombogenicity: preliminary report. Bull N Y Acad Med. 1975;51(8):974-83.

21. Medalie JH, Levene C, Papier C, Goldbourt U, Dreyfuss F, Oron D, et al. Blood groups, myocardial infarction and angina pectoris among 10,000 adult males. N Engl J Med. 1971;285(24):1348-53.

For peer review only - http://bmjopen.bmj.com/site/about/guidelines.xhtml

22. Platt D, Muhlberg W, Kiehl L, Schmitt-Ruth R. ABO blood group system, age, sex, risk factors and cardiac infarction. Arch Gerontol Geriat. 1985;4(3):241-9.

For peer review only

Table 1. Characteristics of study participants.

	Donor cohort		Patient cohort	
Number of subjects	1 164 561		961 637	
Female sex, N (%)	548 234	(47.1)	560 537	(58.3)
Age at start of follow-up, N (%)				
18-40 years	910 537	(78.2)	134 160	(14.0)
41-60 years	246 772	(21.2)	185 080	(19.2)
61-75 years	7 252	(0.6)	307 216	(31.9)
>75 years	0	(0.0)	335 181	(34.9)
Median (IQR)*	28	(22-38)	69	(54-79)
ABO blood group, N (%)				
A	522 364	(44.9)	444 075	(46.2)
AB	62 001	(5.3)	47 610	(5.0)
B	131 751	(11.3)	101 355	(10.5)
O	448 445	(38.5)	368 597	(38.3)
Born in Sweden, N (%)	1 076 286	(92.4)	858 098	(89.2)

*SD denotes standard deviation; IQR denotes interquartile range.

For peer review only

For peer review only - http://bmjopen.bmj.com/site/about/guidelines.xhtml

1

Table 2. Relative risk of aortic dissection or aortic aneurysm in relation to ABO blood group, presented separately for the donor and recipient cohorts.

	Donor cohort		Patient cohort	
ABO blood group	Events/ person-years	Incidence rate ratio (95% confidence interval)	Events/ person-years	Incidence rate ratio (95% confidence interval)
A	2 281/9 344 274	1.00 (0.94-1.06)	7 343/3 911 097	1.02 (0.98-1.05)
AB	268/1 125 603	0.94 (0.83-1.07)	783/ 426 761	1.04 (0.96-1.12)
B	537/2 348 996	0.93 (0.85-1.03)	1 441/ 907 053	0.90 (0.85-0.96)
O	1 977/7 984 924	1.00 (ref)	6 054/3 292 052	1.00 (ref)
Non-O	3 086/12 818 872	0.98 (0.93-1.04)	9 567/5 244 912	1.00 (0.97-1.03)
O	1 977/7 984 924	1.00 (ref)	6 054/3 292 052	1.00 (ref)

2
3
4
5
6

For peer review only

For peer review only - http://bmjopen.bmj.com/site/about/guidelines.xhtml

Table 3. Relative risk of aortic dissection in relation to ABO blood group, presented separately for the donor and recipient cohorts.

	Donor cohort		Patient cohort	
ABO blood group	Events/ person-years	Incidence rate ratio (95% confidence interval)	Events/ person-years	Incidence rate ratio (95% confidence interval)
A	426/9 350 727	0.89 (0.77-1.01)	933/3 927 796	0.98 (0.89-1.08)
AB	55/1 126 318	0.93 (0.70-1.23)	116/ 428 385	1.16 (0.95-1.41)
B	106/2 350 567	0.88 (0.71-1.09)	223/ 910 029	1.05 (0.91-1.22)
O	414/7 990 859	1.00 (ref)	799/3 305 968	1.00 (ref)
Non-O	587/12 827 612	0.89 (0.78-1.01)	1 272/5 266 210	1.01 (0.92-1.10)
O	414/7 990 859	1.00 (ref)	799/3 305 968	1.00 (ref)

For peer review only

Table 4. Relative risk of aortic aneurysm in relation to ABO blood group, presented separately for the donor and recipient cohorts.

ABO blood group	Donor cohort		Patient cohort	
	Events/ person-years	Incidence rate ratio (95% confidence interval)	Events/ person-years	Incidence rate ratio (95% confidence interval)
A	2 056/9 345 277	1.02 (0.96-1.09)	6 852/3 912 179	1.02 (0.98-1.05)
AB	242/1 125 746	0.97 (0.85-1.11)	733/ 426 928	1.04 (0.97-1.13)
B	483/2 349 202	0.95 (0.86-1.05)	1 321/ 907 322	0.89 (0.84-0.94)
O	1 740/7 985 856	1.00 (ref)	5 648/3 292 796	1.00 (ref)
Non-O	2 781/12 820 224	1.00 (0.95-1.07)	8 906/5 246 429	1.00 (0.96-1.03)
O	1 740/7 985 856	1.00 (ref)	5 648/3 292 796	1.00 (ref)

For peer review only - http://bmjopen.bmj.com/site/about/guidelines.xhtml

11

For peer review only

For peer review only - http://bmjopen.bmj.com/site/about/guidelines.xhtml

Supplementary table 1. ICD codes used for group classifications

ICD version	Group	Diagnosis	Description
10	1	I710	Aortic dissection
10	1	I711	Thoracic aortic aneurysm, ruptured
10	1	I713	Abdominal aortic aneurysm, ruptured
10	1	I715	Thoracoabdominal aortic aneurysm, ruptured
10	1	I718	Aortic aneurysm with unspecified location, ruptured
10	1	I712	Thoracic aortic aneurysm, non-ruptured
10	1	I714	Abdominal aortic aneurysm, non-ruptured
10	1	I716	Thoracoabdominal aortic aneurysm, non-ruptured
10	1	I719	Aortic aneurysm with unspecified location, non-ruptured
10	2	I710	Aortic dissection
10	3	I711	Thoracic aortic aneurysm, ruptured
10	3	I713	Abdominal aortic aneurysm, ruptured
10	3	I715	Thoracoabdominal aortic aneurysm, ruptured
10	3	I718	Aortic aneurysm with unspecified location, ruptured
10	3	I712	Thoracic aortic aneurysm, non-ruptured
10	3	I714	Abdominal aortic aneurysm, non-ruptured
10	3	I716	Thoracoabdominal aortic aneurysm, non-ruptured
10	3	I719	Aortic aneurysm with unspecified location, non-ruptured
9	1	4410	Aortic dissection
9	1	4411	Thoracic aortic aneurysm, ruptured
9	1	4413	Abdominal aortic aneurysm, ruptured
9	1	4416	Thoracoabdominal aortic aneurysm, ruptured
9	1	4415	Aortic aneurysm with unspecified location, ruptured
9	1	4412	Thoracic aortic aneurysm, non-ruptured
9	1	4414	Abdominal aortic aneurysm, non-ruptured
9	1	4417	Thoracoabdominal aortic aneurysm, non-ruptured
9	1	4419	Aortic aneurysm with unspecified location, non-ruptured
9	2	4410	Aortic dissection
9	3	4411	Thoracic aortic aneurysm, ruptured
9	3	4413	Abdominal aortic aneurysm, ruptured
9	3	4416	Thoracoabdominal aortic aneurysm, ruptured
9	3	4415	Aortic aneurysm with unspecified location, ruptured
9	3	4412	Thoracic aortic aneurysm, non-ruptured
9	3	4414	Abdominal aortic aneurysm, non-ruptured
9	3	4417	Thoracoabdominal aortic aneurysm, non-ruptured
9	3	4419	Aortic aneurysm with unspecified location, non-ruptured
8	1	4410	Aortic dissection
8	1	4411	Thoracic aortic aneurysm
8	1	4412	Abdominal aortic aneurysm
8	1	4419	Aortic aneurysm with unspecified location, non-ruptured
8	2	4410	Aortic dissection
8	3	4411	Thoracic aortic aneurysm
8	3	4412	Abdominal aortic aneurysm
8	3	4419	Aortic aneurysm with unspecified location, non-ruptured
7	1	45100	Aortic dissection
7	1	45110	Aortic dissection

For peer review only

Supplementary table 2. Group description

Group	Group name	Description
1	Aortic disease	Aortic aneurysm and aortic dissection
2	Aortic dissection	Aortic dissection
3	Aortic aneurysm	Non-ruptured or ruptured aortic aneurysm

For peer review only

STROBE Statement—Checklist of items that should be included in reports of ***cohort studies***

	Item No	Recommendation
**Title and abstract**	1	(*a*) Indicate the study's design with a commonly used term in the title or the abstract Page 1
		(*b*) Provide in the abstract an informative and balanced summary of what was done and what was found Page 2
**Introduction**		
Background/rationale	2	Explain the scientific background and rationale for the investigation being reported Page 4
Objectives	3	State specific objectives, including any prespecified hypotheses Page 4
**Methods**		
Study design	4	Present key elements of study design early in the paper Page 5
Setting	5	Describe the setting, locations, and relevant dates, including periods of recruitment, exposure, follow-up, and data collection Page 5
Participants	6	(*a*) Give the eligibility criteria, and the sources and methods of selection of participants. Describe methods of follow-up Page 5
		(*b*) For matched studies, give matching criteria and number of exposed and unexposed Page 5
Variables	7	Clearly define all outcomes, exposures, predictors, potential confounders, and effect modifiers. Give diagnostic criteria, if applicable Page 6
Data sources/ measurement	8*	For each variable of interest, give sources of data and details of methods of assessment (measurement). Describe comparability of assessment methods if there is more than one group Page 5
Bias	9	Describe any efforts to address potential sources of bias Page 6
Study size	10	Explain how the study size was arrived at NA
Quantitative variables	11	Explain how quantitative variables were handled in the analyses. If applicable, describe which groupings were chosen and why NA
Statistical methods	12	(*a*) Describe all statistical methods, including those used to control for confounding Page 6
		(*b*) Describe any methods used to examine subgroups and interactions Page 6
		(*c*) Explain how missing data were addressed Page 6
		(*d*) If applicable, explain how loss to follow-up was addressed Page 6
		(*e*) Describe any sensitivity analyses Page 6
**Results**		
Participants	13*	(a) Report numbers of individuals at each stage of study—eg numbers potentially eligible, examined for eligibility, confirmed eligible, included in the study, completing follow-up, and analysed Page 8-9
		(b) Give reasons for non-participation at each stage NA
		(c) Consider use of a flow diagram NA
Descriptive data	14*	(a) Give characteristics of study participants (eg demographic, clinical, social) and information on exposures and potential confounders Page 8-9
		(b) Indicate number of participants with missing data for each variable of interest NA
		(c) Summarise follow-up time (eg, average and total amount) Page 8
Outcome data	15*	Report numbers of outcome events or summary measures over time Page 8-9
Main results	16	(*a*) Give unadjusted estimates and, if applicable, confounder-adjusted estimates and their precision (eg, 95% confidence interval). Make clear which confounders were

For peer review only - http://bmjopen.bmj.com/site/about/guidelines.xhtml

		adjusted for and why they were included Page 8-9
		(*b*) Report category boundaries when continuous variables were categorized NA
		(*c*) If relevant, consider translating estimates of relative risk into absolute risk for a meaningful time period NA
Other analyses	17	Report other analyses done—eg analyses of subgroups and interactions, and sensitivity analyses - Supplementary file
**Discussion**		
Key results	18	Summarise key results with reference to study objectives Page 10-12
Limitations	19	Discuss limitations of the study, taking into account sources of potential bias or imprecision. Discuss both direction and magnitude of any potential bias Page 3 and 11
Interpretation	20	Give a cautious overall interpretation of results considering objectives, limitations, multiplicity of analyses, results from similar studies, and other relevant evidence Page 12
Generalisability	21	Discuss the generalisability (external validity) of the study results Page 10-12
**Other information**		
Funding	22	Give the source of funding and the role of the funders for the present study and, if applicable, for the original study on which the present article is based Page 3

*Give information separately for exposed and unexposed groups.

Note: An Explanation and Elaboration article discusses each checklist item and gives methodological background and published examples of transparent reporting. The STROBE checklist is best used in conjunction with this article (freely available on the Web sites of PLoS Medicine at http://www.plosmedicine.org/, Annals of Internal Medicine at http://www.annals.org/, and Epidemiology at http://www.epidem.com/). Information on the STROBE Initiative is available at http://www.strobe-statement.org.

For peer review only

For peer review only - http://bmjopen.bmj.com/site/about/guidelines.xhtml

BMJ Open

ABO blood group and the risk of aortic disease - A nationwide cohort study

Journal:	*BMJ Open*
Manuscript ID	bmjopen-2019-036040.R2
Article Type:	Original research
Date Submitted by the Author:	16-Jul-2020

Complete List of Authors:	Zindovic, Igor; Department of Cardiothoracic Surgery, Clinical Sciences, Lund University, Skane University Hospital<br>Edgren, Gustaf; Karolinska Universitetssjukhuset, Department of Medicine, Solna, Clinical Epidemiology Division, Karolinska Institutet, Stockholm, Sweden; Department of Cardiology, Södersjukhuset, Stockholm, Sweden<br>Nozohoor, Shahab; Department of Cardiothoracic Surgery, Clinical Sciences, Lund University, Skane University Hospital<br>Majeed, Ammar; Department of Medicine, Solna, Clinical Epidemiology Division, Karolinska Institutet, Stockholm, Sweden; Department of Gastroenterology, The Alfred Health, Melbourne, Victoria, Australia

**Primary Subject Heading**:	Epidemiology
Secondary Subject Heading:	Cardiovascular medicine
Keywords:	EPIDEMIOLOGY, Thoracic surgery < SURGERY, Bleeding disorders & coagulopathies < HAEMATOLOGY

SCHOLARONE™ Manuscripts

I, the Submitting Author has the right to grant and does grant on behalf of all authors of the Work (as defined in the below author licence), an exclusive licence and/or a non-exclusive licence for contributions from authors who are: i) UK Crown employees; ii) where BMJ has agreed a CC-BY licence shall apply, and/or iii) in accordance with the terms applicable for US Federal Government officers or employees acting as part of their official duties; on a worldwide, perpetual, irrevocable, royalty-free basis to BMJ Publishing Group Ltd ("BMJ") its licensees and where the relevant Journal is co-owned by BMJ to the co-owners of the Journal, to publish the Work in this journal and any other BMJ products and to exploit all rights, as set out in our licence.

The Submitting Author accepts and understands that any supply made under these terms is made by BMJ to the Submitting Author unless you are acting as an employee on behalf of your employer or a postgraduate student of an affiliated institution which is paying any applicable article publishing charge ("APC") for Open Access articles. Where the Submitting Author wishes to make the Work available on an Open Access basis (and intends to pay the relevant APC), the terms of reuse of such Open Access shall be governed by a Creative Commons licence – details of these licences and which Creative Commons licence will apply to this Work are set out in our licence referred to above.

Other than as permitted in any relevant BMJ Author's Self Archiving Policies, I confirm this Work has not been accepted for publication elsewhere, is not being considered for publication elsewhere and does not duplicate material already published. I confirm all authors consent to publication of this Work and authorise the granting of this licence.

For peer review only

ABO blood group and the risk of aortic disease - A nationwide cohort study

Igor Zindovic MD PhD[1], Gustaf Edgren MD PhD[2,3], Shahab Nozohoor MD PhD[1], Ammar Majeed MD PhD[2,4,5]

[1]Lund University, Skåne University Hospital, Department of Clinical Sciences, Department of Cardiothoracic Surgery, Lund, Sweden; [2]Department of Medicine, Solna, Clinical Epidemiology Division, Karolinska Institutet, Stockholm, Sweden; [3]Department of Cardiology, Södersjukhuset, Stockholm, Sweden; [4]Department of Gastroenterology, The Alfred Health, Melbourne, Victoria, Australia; [5]Central Clinical School, Monash University, Melbourne, Victoria, Australia.

Corresponding author: Igor Zindovic, MD PhD

Department of Cardiothoracic Surgery

Skåne University Hospital, SE-221 85 Lund, Sweden

Phone: +46 46-171000; E-mail: igor.zindovic@med.lu.se

Co-author contact information:

Gustaf.Edgren@ki.se; Shahab.Nozohoor@med.lu.se; Ammar.Majeed@ki.se

For peer review only

ABSTRACT

Objectives: To analyze the association between ABO blood group and aortic disease using data on blood donors and transfused patients from Sweden.

Design: This was a retrospective study using data from the Swedish portion of the Scandinavian Donations and Transfusions database (SCANDAT2). The association between ABO blood group and aortic disease was analyzed using log-linear Poisson regression models and presented as incidence rate ratios (IRR:s).

Setting: Swedish population-based study.

Participants: The study cohort consisted of 1 164 561 Swedish blood donors and 961 637 transfused patients with a combined follow-up time of 29 390 649 person-years.

Primary and secondary outcome measures: IRR:s of aortic events (*i.e.* aortic aneurysms and/or aortic dissections) in relation to patient blood group.

Results: A total of 20 684 aortic events occurred during the study period. Non-O donors and patients had similar incidence of aortic events when compared to blood group O donors and patients with an IRR of 0.98 (95% confidence interval [CI], 0.93-1.04) and 1.00 (95% CI, 0.97-1.03), respectively. There were no differences between non-O and blood group O individuals when aortic dissections and aortic aneurysms were analyzed separately. Blood group B conferred a lower risk of aortic aneurysms in the patient cohort when compared to blood group O (IRR, 0.90; 95% CI, 0.85-0.96).

Conclusions: In the present study, there were no statistically significant associations between ABO blood group and the risk of aortic disease. A possible protective effect of blood group B was observed in the patient cohort but this finding requires further investigation.

For peer review only - http://bmjopen.bmj.com/site/about/guidelines.xhtml

STRENGTHS AND LIMITATIONS OF THE STUDY

- Data were collected from a quality registry covering all blood donations and transfusions 1968 to 2012.
- This study is strengthened by complete follow-up of the study cohort.
- This is by far the largest study assessing the association between blood groups and aortic disease.
- The study is limited by donors representing a selected cohort of healthy individuals, whereas the patient cohort has a higher prevalence of co-morbidities compared to the general population.
- Another limitation is that detailed information on co-morbidities of individuals in the study population was not known.

FUNDING

The SCANDAT2 database was made possible through grants to Gustaf Edgren from the Swedish Research Council (2017-01954) and the Danish Research Council.

COMPETING INTERESTS STATEMENT

The authors have no conflicts of interest to declare.

DATA AVAILABILITY:

No additional data available.

For peer review only

INTRODUCTION

Aortic aneurysm and dissection are potentially life-threatening conditions that share common risk factors—age, hypertension, atherosclerosis, and smoking—with other types of cardiovascular disease (1-3). The association between ABO blood groups and cardiovascular disease and venous thromboembolism (VTE) has been widely reported, but the pathophysiological mechanisms are not fully understood (4-8).

Few studies have examined the association between ABO blood group and aortic disease (9-11). In an analysis of 504 patients with abdominal aortic aneurysm (AAA), Viklander *et. al.* did not find a significant difference in distribution of blood groups between patients with AAA or ruptured AAA compared to a population-based control group (9). Fatic *et. al.* on the other hand, demonstrated that a significantly higher proportion of AAA patients had blood group O compared to a control group consisting of blood donors from the general population of Montenegro (10). Available research is limited by the small size of the studies, conflicting results and the omission of patients with aortic dissection, warranting further examination of the relationship between blood groups and aortic disease.

The aim of the present study was to evaluate the association between ABO blood group and aortic disease using data from a large cohort of Swedish individuals derived from the Scandinavian Donations and Transfusions (SCANDAT2) database (12).

For peer review only

MATERIAL AND METHODS

Data source

The study was based on the Scandinavian Donations and Transfusions database (SCANDAT2). This is a computerized, combined donation and transfusion register using data on more than 1.6 million blood donors and 2 million recipients of blood products in Sweden from 1968 to 2012 and in Denmark from 1981 to 2012 (12, 13). Using the unique personal identity numbers assigned to all residents of the two countries, SCANDAT2 is linked to national population, death, and migration registers, allowing for the complete follow-up of all individuals in the register up to and including December 31, 2012 (14). The database is also linked to the national inpatient, outpatient, cause of death and cancer registries with information on registered diagnoses coded as the International Classification of Diseases (ICD) codes, versions 6-10.

Study Design

This was a retrospective data analysis based on individuals included in the SCANDAT2 register. For the purpose of these analyses, we only used data from the Swedish portion of the SCANDAT2 database and restricted the analysis to donors and patients who donated or received their first blood product after January 1, 1970, when the patient register in Sweden had good national coverage. Donors and recipients with a documented blood group in SCANDAT2, who had given or received at least one blood product between January 1, 1970 and December 31, 2012, were included in the study. We used a set of ICD codes to identify donors and recipients who were diagnosed with either aortic aneurysm or aortic dissection (Supplementary Table 1) based on information from the patient register. The different ICD codes were grouped into 2 different disease groups based on etiological factors or disease characteristics (Supplementary Table 2). Recognizing that aortic aneurysm and dissection share common risk factors and disease characteristics, we created a third group that encompassed all ICD codes for aortic aneurysm and dissection. Donors and recipients who had a diagnosis of aortic aneurysm and/or dissection prior to their first donation or transfusion were excluded from the study. In the present study, we used

For peer review only

For peer review only - http://bmjopen.bmj.com/site/about/guidelines.xhtml

the term "aortic disease" to reflect either aortic aneurysm or aortic dissection and the diagnosis of an aortic aneurysm or aortic dissection as an "aortic event".

Statistical analysis

Recognizing differences in demographics and comorbidities, we created separate cohorts for the donors and the recipients. In the analysis for the donor cohort, we followed the donors from the date of first electronically recorded blood donation in SCANDAT2 or the study start date (January 1, 1970), whichever came last. For the transfusion recipient cohort, individuals were followed from the start of the study (January 1, 1970) or 365 days after their first electronically recorded blood transfusion in SCANDAT2, whichever came last. We delayed the patients' entry into the study by one year after their first transfusion to reduce the risk of reverse causation bias (*i.e.* when the indication for their first transfusion would somehow be related to the subsequent occurrence of an aortic event). All study participants were followed up until either the date of diagnosis of aortic aneurysm or dissection, emigration, death or end of follow-up (December 31, 2012), whichever came first. As an individual could be diagnosed with more than one type of aortic aneurysm or have an aneurysm and dissection diagnosed at different time points during their lifetime, we allowed for the same individual to contribute time at risk and events into more than one disease group based on their diagnoses.

Baseline characteristics are presented as means and standard deviations for continuous variables, and frequencies and percentages for categorical ones. The ratio of the incidence of an aortic aneurysm or dissection in individuals with blood group A, AB or B compared to those with blood group O (the incidence rate ratio) was analyzed using log-linear Poisson regression models. The models included ABO blood group (A, AB, B or O) and were adjusted for age (expressed as natural cubic spline with 5 equally spaced knots), sex (female or male), and calendar year of observation (expressed as natural cubic spline with 5 equally spaced knots). The models thus estimated the number of events (of each type) as

For peer review only - http://bmjopen.bmj.com/site/about/guidelines.xhtml

For peer review only

a function of patient blood group (either parameterized as A, AB, B, or O, or as O vs non-O), patient sex, patient age and calendar period of observation, with the logarithm of time at risk as an offset term (Supplementary Table 3). In alternative analyses, ABO blood groups were instead classified as O or non-O. Interactions between age and blood group and age and sex were also assessed by including interaction terms in the Poisson regression model.

All data processing and statistical analyses were performed with SAS statistical analysis software (version 9.4, SAS Institute, Inc., Cary, NC). All *p*-values below 0.05 were considered statistically significant. The creation of SCANDAT2 and the conduct of this study were approved by the Swedish Ethical Review Authority, the Danish Scientific Ethics Committee and the Danish Data Protection Agency.

Patient and Public Involvement

Apart from contributing with their personal study data, patients were not involved in this study.

For peer review only

RESULTS

Study population and follow-up

The donor cohort consisted of 1 164 561 blood donors with a median follow-up time of 17.9 years, and the patient cohort included 961 637 recipients of blood transfusions with a median follow-up time of 8.9 years. Donors had a median age of 28 years at entry and were less often female (47.1%), whereas patients had a median age of 69 years, and were predominantly female (58.3%). The distribution of the different blood groups was similar in the two cohorts, with blood group O comprising about 38% of each cohort (Table 1).

Overall risk of aortic event

Altogether, there were 716 116 donors with a non-O blood group and 448 445 with blood group O, with a total follow-up time of 12 827 612 and 7 990 859 person-years, respectively. We observed 3 086 events (aortic aneurysm or dissection) in donors with non-O blood group compared to 1 977 events in donors with blood group O. The resulting incidence rate ratio (IRR) for any aortic event in donors with a non-O blood group compared to blood group O was 0.98 (95% confidence interval [CI], 0.93-1.04) (Table 2). The patient cohort included 593 040 patients with a non-O blood group and 368 597 patients with blood group O with a combined follow-up time of 5 266 210 and 3 305 968 person-years, respectively. Throughout follow-up, an aortic event (aortic aneurysm or dissection) was diagnosed in 9 567 patients with a non-O blood compared to 6 054 events in those with blood group O. The IRR for such events in patients with non-O blood was 1.00 (95% CI, 0.97-1.03) compared to those with blood group O.

No significant differences were observed when blood groups A, B, AB were compared with blood group O except for blood group B having an IRR 0.90 (95% CI, 0.85-0.96) for the occurrence of any aortic event in the patient cohort (Tables 2-4).

Risk of aortic dissection

Aortic dissection occurred in 587 non-O donors and 414 O donors during the study period,

For peer review only - http://bmjopen.bmj.com/site/about/guidelines.xhtml

For peer review only

corresponding to an IRR of 0.89 (95% CI, 0.78-1.01) for non-O donors compared to blood group O donors (Table 3). Aortic dissection was observed in 1 272 patients with non-O blood and 799 patients with blood group O, with an IRR of 1.01 (95% CI, 0.92-1.10). There were no differences in risk of aortic dissection in patients with blood groups A, B or AB compared to those with blood group O (Table 3).

Risk of aortic aneurysm

Aortic aneurysm was diagnosed in 2 781 donors with non-O blood and 1 740 O donors, resulting in an IRR of 1.00 (95% CI, 0.95-1.07) for non-O donors compared to O donors (Table 4). For the patient cohort, the corresponding numbers were 8 906 and 5 648, respectively, resulting in an IRR of 1.00 (95% CI, 0.96-1.03). Similar results were obtained in the analysis of the risk of aortic aneurysm in patients with blood groups A and AB. However, patients with blood group B had a significantly lower risk of experiencing an aortic event when compared to patients with blood group O with an IRR of 0.89 (95% CI, 0.84-0.94) (Table 2).

For peer review only

DISCUSSION

Here, we present results from the hitherto largest study of the association between ABO blood groups and aortic aneurysm or dissection with more than two million Swedish blood donors and transfusion recipients, a total of 20 684 aortic events and a combined follow-up time of almost 30 million person-years. The present study did not demonstrate a statistically significant association between ABO blood group and aortic disease with the exception of an inconsistent association in the patient cohort between blood group B and aortic aneurysm.

Previously, Fatic *et. al.* showed that a higher proportion of patients diagnosed with AAA had blood group O when compared to blood donors in the normal population (11), whereas Viklander *et. al.* could not find any association between blood groups and the risk of developing AAA or AAA rupture (10). It has been reported that non-O blood groups (*i.e.* blood groups A, B and AB) are associated with significantly higher risk of venous thromboembolism and cardiovascular disease, including myocardial infarction, peripheral vascular disease and cerebrovascular stroke (4-8). This could, in part, be explained by blood group O patients having 25-30% lower levels of circulating Factor VIII (FVIII) and von Willebrand factor (VWF) (15). However, Dentali *et. al.* demonstrated that non-O patients were at an increased risk of VTE even after adjustment for different levels of FVIII/VWF. Furthermore, it has been shown that that the genetic variation of ABO is associated with atherosclerosis and biomarkers of vascular inflammation (16), both of which are recognized risk factors for aortic aneurysm formation and aortic dissection (1, 17). The normal physiology of the vascular system relies on a negatively charged vascular wall, which repels the similarly negatively charged red blood cells and many negatively charged circulating proteins. A reduction of the negative charge may make the vascular wall more prone to thrombus formation and may also have an effect on the transportation of charged circulating proteases and cytokines into the vessel wall (18, 19). ABO blood groups and Rhesus antigen status have both been shown to influence the size of the charge potential (20), but the findings of the present study indicate that the previously described pathophysiological mechanisms of cardiovascular disease associated with ABO blood groups do not seem to translate to the formation of aortic aneurysms or the development of aortic dissection.

For peer review only - http://bmjopen.bmj.com/site/about/guidelines.xhtml

The differences in risk of cardiovascular disease between non-O and O individuals has previously been suggested as a specific effect of blood group A (21, 22). The analyses of different ABO blood groups in the present study could not demonstrate a specific effect of blood group A. Instead, our analyses showed a protective effect of blood group B on aortic disease in the patient cohort, primarily driven by significantly lower risk of aortic aneurysm development. To our knowledge, there are no previous reports suggesting an association between blood group B and cardiovascular disease and, thus, we have no plausible pathophysiological explanation for the specific effect of blood group B in this study. Furthermore, this association was not demonstrated in the donor cohort and, therefore, our speculation is that the protective effect of blood group B on the incidence of aortic disease might have been a chance finding.

The results presented in this study need some careful consideration. First, the donor and the patient cohort both represent selected populations and not the general population. However, to our knowledge, there are no data on blood group distributions in large cohorts of randomly selected individuals and therefore, donors or patients have generally served as control groups in similar studies in the past (4, 5, 6, 7, 8, 9, 12, 13). Donors represent a selected cohort of healthy individuals, whereas the patient cohort has a higher prevalence of co-morbidities compared to the general population. Despite the differences in health profile, this study did not demonstrate any major differences in blood group distribution between these two groups and therefore, we have no reason to believe that the distributions in donors and patients are not reflective of those in the general population. The external validity of the study might, therefore, be affected. Aortic aneurysm and dissection generally affect individuals older than the average age of blood donors. However, we did not find a significant interaction between ABO blood group and age. Furthermore, The SCANDAT2 database does not include patient genotyping and, therefore, we cannot fully assess the distribution of O alleles in blood group A and B patients. The use of hospital registers carries the inherent risk of misclassification, but it is unlikely that it relates to patient blood group. The limitations of the ICD diagnosis classification system did not permit us to definitively separate non-ruptured from ruptured aortic aneurysms and, thus, these were analyzed together, despite

For peer review only - http://bmjopen.bmj.com/site/about/guidelines.xhtml

ruptured aortic aneurysms potentially having larger similarities with aortic dissections. This should not, however, influence results from the analyses of the combination of aortic aneurysm and dissection.

On the other hand, there are several strengths to this study. It is by far the largest study investigating the association between ABO blood groups and aortic disease. The use of nationwide population and health registers allowed for complete follow-up and adds to the strength of the study. Blood group data was recorded prospectively and has high validity. Even though patients are potentially aware of their blood group, it is unlikely that such awareness has influenced their behavior. The prevalence of other risk factors for aortic disease should be similar between the different blood groups and, thereby, it should not have a confounding effect on the results of the analyses.

In conclusion, this large study did not demonstrate a significant association between ABO blood group and the risk of aortic disease. A possible protective effect of blood group B was, however observed in the patient cohort. Further studies are needed to confirm the presence and strength of this association.

Authorship statement

The conception and design of the study was performed by Igor Zindovic, Gustaf Edgren and Ammar Majeed. Gustaf Edgren and Ammar Majeed performed the statistical analyses and Igor Zindovic and Shahab Nozohoor produced the first draft of the manuscript. The data was interpreted by all co-authors. The manuscript was critically reviewed for important intellectual content and the final version was approved by all authors. All authors had full access to all the data (including statistical reports and tables) of the study and can take responsibility for the integrity of the data and the accuracy of the data analysis.

WORD COUNT

3779

For peer review only - http://bmjopen.bmj.com/site/about/guidelines.xhtml

REFERENCES

1. Landenhed M, Engstrom G, Gottsater A, Caulfield MP, Hedblad B, Newton-Cheh C, et al. Risk profiles for aortic dissection and ruptured or surgically treated aneurysms: a prospective cohort study. J Am Heart Assoc. 2015;4(1):e001513.

2. Hagan PG, Nienaber CA, Isselbacher EM, Bruckman D, Karavite DJ, Russman PL, et al. The International Registry of Acute Aortic Dissection (IRAD): new insights into an old disease. JAMA. 2000;283(7):897-903.

3. Dahlof B. Cardiovascular disease risk factors: epidemiology and risk assessment. Am J Cardiol. 2010;105(1 Suppl):3A-9A.

4. Vasan SK, Rostgaard K, Majeed A, Ullum H, Titlestad KE, Pedersen OB, et al. ABO Blood Group and Risk of Thromboembolic and Arterial Disease: A Study of 1.5 Million Blood Donors. Circulation. 2016;133(15):1449-57; discussion 57.

5. Dentali F, Sironi AP, Ageno W, Crestani S, Franchini M. ABO blood group and vascular disease: an update. Semin Thromb Hemost. 2014;40(1):49-59.

6. Dentali F, Sironi AP, Ageno W, Turato S, Bonfanti C, Frattini F, et al. Non-O blood type is the commonest genetic risk factor for VTE: results from a meta-analysis of the literature. Semin Thromb Hemost. 2012;38(5):535-48.

7. Franchini M, Favaloro EJ, Targher G, Lippi G. ABO blood group, hypercoagulability, and cardiovascular and cancer risk. Crit Rev Clin Lab Sci. 2012;49(4):137-49.

8. Wu O, Bayoumi N, Vickers MA, Clark P. ABO(H) blood groups and vascular disease: a systematic review and meta-analysis. J Thromb Haemost. 2008;6(1):62-9.

9. Viklander G, Wallinder J, Henriksson AE. ABO blood groups and abdominal aortic aneurysm. Transfus Apher Sci. 2012;47(3):351-3.

10. Fatic N, Lukac H, Radojevic N, Simanic I, Banzic I, Pajovic B. O blood group as an indicator for abdominal aortic aneurysm. Eur Rev Med Pharmacol Sci. 2015;19(16):2997-3000.

11. Fatic N, Nikolic A, Vukmirovic M, Radojevic N, Zornic N, Banzic I, et al. Blood groups and acute aortic dissection type III. Arch Med Sci. 2017;13(3):597-600.

For peer review only

For peer review only - http://bmjopen.bmj.com/site/about/guidelines.xhtml

12. Edgren G, Rostgaard K, Vasan SK, Wikman A, Norda R, Pedersen OB, et al. The new Scandinavian Donations and Transfusions database (SCANDAT2): a blood safety resource with added versatility. Transfusion. 2015;55(7):1600-6.

13. Edgren G, Hjalgrim H, Tran TN, Rostgaard K, Shanwell A, Titlestad K, et al. A population-based binational register for monitoring long-term outcome and possible disease concordance among blood donors and recipients. Vox sanguinis. 2006;91(4):316-23.

14. Ludvigsson JF, Otterblad-Olausson P, Pettersson BU, Ekbom A. The Swedish personal identity number: possibilities and pitfalls in healthcare and medical research. Eur J Epidemiol. 2009;24(11):659-67.

15. Jenkins PV, O'Donnell JS. ABO blood group determines plasma von Willebrand factor levels: a biologic function after all? Transfusion. 2006;46(10):1836-44.

16. Larson NB, Bell EJ, Decker PA, Pike M, Wassel CL, Tsai MY, et al. ABO blood group associations with markers of endothelial dysfunction in the Multi-Ethnic Study of Atherosclerosis. Atherosclerosis. 2016;251:422-9.

17. Stone JR, Bruneval P, Angelini A, Bartoloni G, Basso C, Batoroeva L, et al. Consensus statement on surgical pathology of the aorta from the Society for Cardiovascular Pathology and the Association for European Cardiovascular Pathology: I. Inflammatory diseases. Cardiovasc Pathol. 2015;24(5):267-78.

18. Srinivasan S, Sawyer PN. Role of surface charge of the blood vessel wall, blood cells, and prosthetic materials in intravascular thrombosis. J Colloid Interface Sci. 1970;32(3):456-63.

19. Michel JB, Jondeau G, Milewicz DM. From genetics to response to injury: vascular smooth muscle cells in aneurysms and dissections of the ascending aorta. Cardiovasc Res. 2018;114(4):578-89.

20. Thubrikar MJ, Cadoff I, Youdin M, Reich T. Effect of blood types and vessel wall surface charge on thrombogenicity: preliminary report. Bull N Y Acad Med. 1975;51(8):974-83.

21. Medalie JH, Levene C, Papier C, Goldbourt U, Dreyfuss F, Oron D, et al. Blood groups, myocardial infarction and angina pectoris among 10,000 adult males. N Engl J Med. 1971;285(24):1348-53.

For peer review only

For peer review only - http://bmjopen.bmj.com/site/about/guidelines.xhtml

22. Platt D, Muhlberg W, Kiehl L, Schmitt-Ruth R. ABO blood group system, age, sex, risk factors and cardiac infarction. Arch Gerontol Geriat. 1985;4(3):241-9.

For peer review only

For peer review only - http://bmjopen.bmj.com/site/about/guidelines.xhtml

Table 1. Characteristics of study participants.

	Donor cohort		Patient cohort	
Number of subjects	1 164 561		961 637	
Female sex, N (%)	548 234	(47.1)	560 537	(58.3)
Age at start of follow-up, N (%)				
18-40 years	910 537	(78.2)	134 160	(14.0)
41-60 years	246 772	(21.2)	185 080	(19.2)
61-75 years	7 252	(0.6)	307 216	(31.9)
>75 years	0	(0.0)	335 181	(34.9)
Median (IQR)*	28	(22-38)	69	(54-79)
ABO blood group, N (%)				
A	522 364	(44.9)	444 075	(46.2)
AB	62 001	(5.3)	47 610	(5.0)
B	131 751	(11.3)	101 355	(10.5)
O	448 445	(38.5)	368 597	(38.3)
Born in Sweden, N (%)	1 076 286	(92.4)	858 098	(89.2)

*SD denotes standard deviation; IQR denotes interquartile range.

For peer review only

Table 2. Relative risk of aortic dissection or aortic aneurysm in relation to ABO blood group, presented separately for the donor and recipient cohorts.

	Donor cohort		Patient cohort	
ABO blood group	Events/ person-years	Incidence rate ratio (95% confidence interval)	Events/ person-years	Incidence rate ratio (95% confidence interval)
A	2 281/9 344 274	1.00 (0.94-1.06)	7 343/3 911 097	1.02 (0.98-1.05)
AB	268/1 125 603	0.94 (0.83-1.07)	783/ 426 761	1.04 (0.96-1.12)
B	537/2 348 996	0.93 (0.85-1.03)	1 441/ 907 053	0.90 (0.85-0.96)
O	1 977/7 984 924	1.00 (ref)	6 054/3 292 052	1.00 (ref)
Non-O	3 086/12 818 872	0.98 (0.93-1.04)	9 567/5 244 912	1.00 (0.97-1.03)
O	1 977/7 984 924	1.00 (ref)	6 054/3 292 052	1.00 (ref)

For peer review only

For peer review only - http://bmjopen.bmj.com/site/about/guidelines.xhtml

Table 3. Relative risk of aortic dissection in relation to ABO blood group, presented separately for the donor and recipient cohorts.

	**Donor cohort**		**Patient cohort**	
ABO blood group	Events/ person-years	Incidence rate ratio (95% confidence interval)	Events/ person-years	Incidence rate ratio (95% confidence interval)
A	426/9 350 727	0.89 (0.77-1.01)	933/3 927 796	0.98 (0.89-1.08)
AB	55/1 126 318	0.93 (0.70-1.23)	116/ 428 385	1.16 (0.95-1.41)
B	106/2 350 567	0.88 (0.71-1.09)	223/ 910 029	1.05 (0.91-1.22)
O	414/7 990 859	1.00 (ref)	799/3 305 968	1.00 (ref)
Non-O	587/12 827 612	0.89 (0.78-1.01)	1 272/5 266 210	1.01 (0.92-1.10)
O	414/7 990 859	1.00 (ref)	799/3 305 968	1.00 (ref)

For peer review only - http://bmjopen.bmj.com/site/about/guidelines.xhtml

Table 4. Relative risk of aortic aneurysm in relation to ABO blood group, presented separately for the donor and recipient cohorts.

ABO blood group	Donor cohort		Patient cohort	
	Events/ person-years	Incidence rate ratio (95% confidence interval)	Events/ person-years	Incidence rate ratio (95% confidence interval)
A	2 056/9 345 277	1.02 (0.96-1.09)	6 852/3 912 179	1.02 (0.98-1.05)
AB	242/1 125 746	0.97 (0.85-1.11)	733/ 426 928	1.04 (0.97-1.13)
B	483/2 349 202	0.95 (0.86-1.05)	1 321/ 907 322	0.89 (0.84-0.94)
O	1 740/7 985 856	1.00 (ref)	5 648/3 292 796	1.00 (ref)
Non-O	2 781/12 820 224	1.00 (0.95-1.07)	8 906/5 246 429	1.00 (0.96-1.03)
O	1 740/7 985 856	1.00 (ref)	5 648/3 292 796	1.00 (ref)

For peer review only

For peer review only - http://bmjopen.bmj.com/site/about/guidelines.xhtml

11

For peer review only - http://bmjopen.bmj.com/site/about/guidelines.xhtml

Supplementary table 1. ICD codes used for group classifications

ICD version	Group	Diagnosis	Description
10	1	I710	Aortic dissection
10	1	I711	Thoracic aortic aneurysm, ruptured
10	1	I713	Abdominal aortic aneurysm, ruptured
10	1	I715	Thoracoabdominal aortic aneurysm, ruptured
10	1	I718	Aortic aneurysm with unspecified location, ruptured
10	1	I712	Thoracic aortic aneurysm, non-ruptured
10	1	I714	Abdominal aortic aneurysm, non-ruptured
10	1	I716	Thoracoabdominal aortic aneurysm, non-ruptured
10	1	I719	Aortic aneurysm with unspecified location, non-ruptured
10	2	I710	Aortic dissection
10	3	I711	Thoracic aortic aneurysm, ruptured
10	3	I713	Abdominal aortic aneurysm, ruptured
10	3	I715	Thoracoabdominal aortic aneurysm, ruptured
10	3	I718	Aortic aneurysm with unspecified location, ruptured
10	3	I712	Thoracic aortic aneurysm, non-ruptured
10	3	I714	Abdominal aortic aneurysm, non-ruptured
10	3	I716	Thoracoabdominal aortic aneurysm, non-ruptured
10	3	I719	Aortic aneurysm with unspecified location, non-ruptured
9	1	4410	Aortic dissection
9	1	4411	Thoracic aortic aneurysm, ruptured
9	1	4413	Abdominal aortic aneurysm, ruptured
9	1	4416	Thoracoabdominal aortic aneurysm, ruptured
9	1	4415	Aortic aneurysm with unspecified location, ruptured
9	1	4412	Thoracic aortic aneurysm, non-ruptured
9	1	4414	Abdominal aortic aneurysm, non-ruptured
9	1	4417	Thoracoabdominal aortic aneurysm, non-ruptured
9	1	4419	Aortic aneurysm with unspecified location, non-ruptured
9	2	4410	Aortic dissection
9	3	4411	Thoracic aortic aneurysm, ruptured
9	3	4413	Abdominal aortic aneurysm, ruptured
9	3	4416	Thoracoabdominal aortic aneurysm, ruptured
9	3	4415	Aortic aneurysm with unspecified location, ruptured
9	3	4412	Thoracic aortic aneurysm, non-ruptured
9	3	4414	Abdominal aortic aneurysm, non-ruptured

For peer review only

9	3	4417	Thoracoabdominal aortic aneurysm, non-ruptured
9	3	4419	Aortic aneurysm with unspecified location, non-ruptured
8	1	4410	Aortic dissection
8	1	4411	Thoracic aortic aneurysm
8	1	4412	Abdominal aortic aneurysm
8	1	4419	Aortic aneurysm with unspecified location, non-ruptured
8	2	4410	Aortic dissection
8	3	4411	Thoracic aortic aneurysm
8	3	4412	Abdominal aortic aneurysm
8	3	4419	Aortic aneurysm with unspecified location, non-ruptured
7	1	45100	Aortic dissection
7	1	45110	Aortic dissection

Supplementary table 2. Group description

Group	Group name	Description
1	Aortic disease	Aortic aneurysm and aortic dissection
2	Aortic dissection	Aortic dissection
3	Aortic aneurysm	Non-ruptured or ruptured aortic aneurysm

For peer review only

4

Supplementary table 3. Regression estimates and corresponding Incidence Rate Ratios, presented separately for the donor and recipient cohorts.

Group	Variable	Donors' Estimates			Patients' Estimates		
		Regression Estimate	Standard Error	IRR (95%CI)	Regression Estimate	Standard Error	IRR (95%CI)
Aneurysm and Dissection							
	Intercept	-88.131	70.894		-890.153	167.331	
	Blood Group						
	A	0.003	0.031	1.00 (0.94-1.06)	0.018	0.017	1.02 (0.98-1.05)
	AB	-0.058	0.065	0.94 (0.83-1.07)	0.031	0.038	1.04 (0.96-1.12)
	B	-0.072	0.049	0.93 (0.85-1.03)	-0.095	0.029	0.90 (0.85-0.96)
	O	0.000	.	1.00 (.-.)	0.000	.	1.00 (.-.)
	Non-o	-0.016	0.029	0.98 (0.93-1.04)	0.001	0.016	1.00(0.97-1.03)
	Sex						
	Female	1.250	0.040	3.49 (3.23-3.78)	1.115	0.017	3.05(2.95-3.15)
	Male	0.000	.	1.00 (.-.)	0.000	.	1.00 (.-.)
	Age (spline)						
	Level 1	0.000	.	1.00 (.-.)	0.000	.	1.00 (.-.)
	Level 2	0.088	0.010	1.09 (1.07-1.11)	0.033	0.011	1.03(1.01-1.06)
	Level 3	0.003	0.001	1.00 (1.00-1.00)	0.004	0.001	1.00(1.00-1.01)
	Level 4	-0.004	0.002	1.00 (0.99-1.00)	-0.006	0.002	0.99(0.99-1.00)
	Level 5	-0.003	0.001	1.00 (0.99-1.00)	-0.001	0.001	1.00(1.00-1.00)

For peer review only

	Year (spline)						
	Level 1	0.000	.	1.00 (.-.)	0.000	.	1.00 (.-.)
	Level 2	0.037	0.036	1.04 (0.97-1.11)	0.443	0.084	1.56(1.32-1.84)
	Level 3	-0.016	0.007	0.98 (0.97-1.00)	-0.041	0.010	0.96(0.94-0.98)
	Level 4	0.053	0.014	1.05 (1.03-1.08)	0.075	0.017	1.08(1.04-1.11)
	Level 5	-0.057	0.010	0.94 (0.93-0.96)	-0.047	0.009	0.95(0.94-0.97)
Aortic Dissection							
	Intercept	-59.428	114.105		-1112.308	397.726	
	Blood Group						
	A	-0.131	0.071	0.89 (0.77-1.01)	-0.052	0.047	0.98 (0.89-1.08)
	AB	-0.128	0.150	0.93 (0.70-1.23)	0.146	0.097	1.16 (0.95-1.41)
	B	-0.123	0.111	0.88 (0.71-1.09)	0.059	0.074	1.05 (0.91-1.22)
	O	0.000	.	1.00 (.-.)	0.000	.	1.00 (.-.)
	Non-o	-0.129	0.066	0.89 (0.78-1.01)	-0.016	0.044	1.01 (0.92-1.10)
	Sex						
	Female	0.000	.	1.00 (.-.)	0.000	.	1.00 (.-.)
	Male	0.956	0.083	2.60 (2.21-3.06)	0.745	0.045	2.11(1.93-2.30)
	Age (spline)						
	Level 1	0.000	.	1.00 (.-.)	0.000	.	1.00 (.-.)
	Level 2	0.135	0.019	1.14 (1.10-1.19)	0.041	0.019	1.04(1.00-1.08)
	Level 3	-0.002	0.002	1.00 (0.99-1.00)	0.001	0.002	1.00(1.00-1.00)

For peer review only

	Level 4	0.004	0.004	1.00 (1.00-1.01)	-0.002	0.003	1.00(0.99-1.00)
	Level 5	-0.004	0.003	1.00 (0.99-1.00)	-0.001	0.002	1.00(0.99-1.00)
	Year (spline)						
	Level 1	0.000	.	1.00 (.-.)	0.000	.	1.00 (.-.)
	Level 2	0.021	0.058	1.02 (0.91-1.14)	0.554	0.201	1.74(1.18-2.58)
	Level 3	-0.017	0.011	0.98 (0.96-1.01)	-0.056	0.024	0.95(0.90-0.99)
	Level 4	0.058	0.024	1.06 (1.01-1.11)	0.098	0.041	1.10(1.02-1.20)
	Level 5	-0.067	0.018	0.94 (0.90-0.97)	-0.058	0.020	0.94(0.91-0.98)
Aortic Aneurysm							
	Intercept	-139.788	91.425		-861.716	185.020	
	Blood Group						
	A	0.029	0.033	1.02 (0.96-1.09)	0.020	0.018	1.02 (0.98-1.05)
	AB	-0.033	0.069	0.97 (0.85-1.11)	0.030	0.039	1.04 (0.97-1.13)
	B	-0.048	0.051	0.95 (0.86-1.05)	-0.114	0.030	0.89 (0.84-0.94)
	O	0.000	.	1.00 (.-.)	0.000	.	1.00 (.-.)
	Non-o	0.010	0.031	1.00 (0.95-1.07)	0.000	0.017	1.00(0.96-1.03)
	Sex						
	Female	1.299	0.043	3.67 (3.37-3.99)	1.158	0.018	3.18(3.07-3.30)
	Male	0.000	.	1.00 (.-.)	0.000	.	1.00 (.-.)
	Age (spline)						
	Level 1	0.000	.	1.00 (.-.)	0.000	.	1.00 (.-.)
	Level 2	0.074	0.011	1.08 (1.05-1.10)	0.035	0.013	1.04(1.01-1.06)

For peer review only

Level 3	0.004	0.001	1.00 (1.00-1.01)	0.004	0.001	1.00(1.00-1.01)
Level 4	-0.007	0.002	0.99 (0.99-1.00)	-0.007	0.002	0.99(0.99-1.00)
Level 5	-0.001	0.001	1.00 (1.00-1.00)	0.000	0.001	1.00(1.00-1.00)
Year (spline)						
Level 1	0.000	.	1.00 (.-.)	0.000	.	1.00 (.-.)
Level 2	0.063	0.046	1.06 (0.97-1.17)	0.428	0.093	1.53(1.28-1.84)
Level 3	-0.019	0.008	0.98 (0.97-1.00)	-0.038	0.011	0.96(0.94-0.98)
Level 4	0.058	0.017	1.06 (1.03-1.09)	0.071	0.019	1.07(1.03-1.11)
Level 5	-0.062	0.011	0.94 (0.92-0.96)	-0.047	0.009	0.95(0.94-0.97)

For peer review only

STROBE Statement—Checklist of items that should be included in reports of ***cohort studies***

	Item No	Recommendation
**Title and abstract**	1	(*a*) Indicate the study's design with a commonly used term in the title or the abstract Page 1
		(*b*) Provide in the abstract an informative and balanced summary of what was done and what was found Page 2
**Introduction**		
Background/rationale	2	Explain the scientific background and rationale for the investigation being reported Page 4
Objectives	3	State specific objectives, including any prespecified hypotheses Page 4
**Methods**		
Study design	4	Present key elements of study design early in the paper Page 5
Setting	5	Describe the setting, locations, and relevant dates, including periods of recruitment, exposure, follow-up, and data collection Page 5
Participants	6	(*a*) Give the eligibility criteria, and the sources and methods of selection of participants. Describe methods of follow-up Page 5
		(*b*) For matched studies, give matching criteria and number of exposed and unexposed Page 5
Variables	7	Clearly define all outcomes, exposures, predictors, potential confounders, and effect modifiers. Give diagnostic criteria, if applicable Page 6
Data sources/ measurement	8*	For each variable of interest, give sources of data and details of methods of assessment (measurement). Describe comparability of assessment methods if there is more than one group Page 5
Bias	9	Describe any efforts to address potential sources of bias Page 6
Study size	10	Explain how the study size was arrived at NA
Quantitative variables	11	Explain how quantitative variables were handled in the analyses. If applicable, describe which groupings were chosen and why NA
Statistical methods	12	(*a*) Describe all statistical methods, including those used to control for confounding Page 6
		(*b*) Describe any methods used to examine subgroups and interactions Page 6
		(*c*) Explain how missing data were addressed Page 6
		(*d*) If applicable, explain how loss to follow-up was addressed Page 6
		(*e*) Describe any sensitivity analyses Page 6
**Results**		
Participants	13*	(a) Report numbers of individuals at each stage of study—eg numbers potentially eligible, examined for eligibility, confirmed eligible, included in the study, completing follow-up, and analysed Page 8-9
		(b) Give reasons for non-participation at each stage NA
		(c) Consider use of a flow diagram NA
Descriptive data	14*	(a) Give characteristics of study participants (eg demographic, clinical, social) and information on exposures and potential confounders Page 8-9
		(b) Indicate number of participants with missing data for each variable of interest NA
		(c) Summarise follow-up time (eg, average and total amount) Page 8
Outcome data	15*	Report numbers of outcome events or summary measures over time Page 8-9
Main results	16	(*a*) Give unadjusted estimates and, if applicable, confounder-adjusted estimates and their precision (eg, 95% confidence interval). Make clear which confounders were

For peer review only - http://bmjopen.bmj.com/site/about/guidelines.xhtml

		adjusted for and why they were included Page 8-9
		(*b*) Report category boundaries when continuous variables were categorized NA
		(*c*) If relevant, consider translating estimates of relative risk into absolute risk for a meaningful time period NA
Other analyses	17	Report other analyses done—eg analyses of subgroups and interactions, and sensitivity analyses - Supplementary file
**Discussion**		
Key results	18	Summarise key results with reference to study objectives Page 10-12
Limitations	19	Discuss limitations of the study, taking into account sources of potential bias or imprecision. Discuss both direction and magnitude of any potential bias Page 3 and 11
Interpretation	20	Give a cautious overall interpretation of results considering objectives, limitations, multiplicity of analyses, results from similar studies, and other relevant evidence Page 12
Generalisability	21	Discuss the generalisability (external validity) of the study results Page 10-12
**Other information**		
Funding	22	Give the source of funding and the role of the funders for the present study and, if applicable, for the original study on which the present article is based Page 3

*Give information separately for exposed and unexposed groups.

Note: An Explanation and Elaboration article discusses each checklist item and gives methodological background and published examples of transparent reporting. The STROBE checklist is best used in conjunction with this article (freely available on the Web sites of PLoS Medicine at http://www.plosmedicine.org/, Annals of Internal Medicine at http://www.annals.org/, and Epidemiology at http://www.epidem.com/). Information on the STROBE Initiative is available at http://www.strobe-statement.org.

For peer review only
